# Toward Low-Cost All-Organic and Biodegradable Li-Ion Batteries

**DOI:** 10.1038/s41598-020-60633-y

**Published:** 2020-03-02

**Authors:** N. Delaporte, G. Lajoie, S. Collin-Martin, K. Zaghib

**Affiliations:** Hydro-Québec, Center of Excellence in Transportation Electrification and Energy Storage, Varennes, Québec J0L 1N0 Canada

**Keywords:** Chemistry, Energy science and technology, Engineering, Materials science

## Abstract

This work presents an alternative method for fabricating Li-ion electrodes in which the use of aluminum/copper current collectors and expensive binders is avoided. Low-cost natural cellulose fibers with a 2-mm length are employed as binder and support for the electrode. The objective of this method is to eliminate the use of heavy and inactive current collector foils as substrates and to replace conventional costly binders with cellulose fibers. Moreover, no harmful solvents, such as N-methylpyrrolidone, are employed for film fabrication. Water-soluble carbons are also utilized to reduce the preparation time and to achieve a better repartition of carbon in the electrode, thus improving the electrochemical performance. Flexible and resistant LiFePO_4_ (LFP), Li_4_Ti_5_O_12_ (LTO), organic 3,4,9,10-perylenetetracarboxylic dianhydride (PTCDA), and graphite electrodes are obtained with active mass loadings similar to those obtained by the current casting method. The initial discharge capacity of approximately 130 mAh·g^−1^ at 2 C is obtained for an LFP/LTO paper battery with an approximately 91.6% capacity retention after 1000 cycles. An all-organic prelithiated PTCDA/graphite cell without a transition metal is prepared and electrochemically tested. It is one of the first self-standing batteries that is composed of organic redox active molecules and biodegradable components reported in literature.

## Introduction

Over the past decade, flexible electronics^1,2^ have undergone rapid developments in the fields of wearable^3,4^ and implantable devices^5^, flexible displays^6^, and even flexible radio frequency identification tags^7^. Li-ion battery is the most developed energy supply technology that satisfies high demands in energy, power, and long cycle life^8,9^. For these reasons, flexible Li-ion batteries have gained particular attention as a future energy storage solution for lightweight and flexible devices^10,11^. The widely used method for fabricating Li-ion electrodes (i.e., web-coating method), however, is unable to adequately satisfy these requirements^12^. In fact, Cu and Al metal foils, which are generally used as current collector and support to spread the cathode or anode ink, lose contact with the active material during repeated bending^13^. Moreover, the conventional method usually employs costly binders (such as polyvinylidene fluoride (PVDF)) that are dissolved in solvents (e.g., N-methyl-2-pyrrolidone (NMP)), which are characterized by high toxicity^14^. Drying such electrodes also consumes a significant amount of energy because of their high boiling point (204.3 °C) and low vapor pressure (e.g., 1 mm Hg at 40 °C for NMP)^15^. A greener process that employs sodium carboxymethyl cellulose as a low-cost binder and water as solvent has been reported^16–18^; however, some major problems have to be resolved. Water-based electrodes exhibit agglomeration effects, poor ink homogeneity, and residual moisture after drying, which considerably reduce the cycle life of the battery^19^. According to the cost modeling proposed by Wood *et al*.^20^, the composite electrode materials and current collectors account for practically 50% of the cost of a Li-ion battery. Furthermore, to avoid corrosion problems that result from contact with electrolytes^21^, the use of a current collector, especially an Al foil, significantly reduces the battery’s energy density. For instance, the Al current collector represents 40% of the total mass of an LFP electrode with an active material loading of approximately 6 mg·cm^−2^.

To resolve the foregoing problems, free-standing electrode films are proposed as a low-cost alternative to fabricate flexible Li-ion electrodes. Free-standing films are generally fabricated according to a paper-making process that is well-known and appropriate for producing substantial amounts of electrodes. Recently, paper^10^, textile^22^, and other organic substrates^23–25^ are reported to be potential components for flexible electrodes because of their good flexibility; however, they cannot be used alone because of their poor electronic conductivity. In view this, self-standing films made of two-dimensional (2D) or unidimensional (1D) carbons have been developed in parallel. These afford a high electronic conductivity and a strong network mainly because of the π-stacking effect (2D carbon) or the interlacing of carbon fibers (1D carbon). For instance, flexible LFP and LTO electrodes made of graphene as a 2D carbon are reported by Shi *et al*.^26^. Alternatively, the use of 1D carbons, such as carbon nanotubes (CNT) or vapor-grown carbon fibers (VGCF) is also proposed to generate a solid 3D network in which the porosity is filled with redox active materials^27,28^. The cost of such electrodes, however, limits the scale-up of this fabrication method. To maintain good electronic conductivity without affecting film integrity, a mixture of cellulose fibers with 2D or 1D carbons are often reported in literature as a less expensive option for fabricating self-standing electrode films. Cornell *et al*. fabricated an LFP composite electrode made of 1D polyacrylonitrile carbon fibers (CF) and TEMPO-oxidized cellulose nanofibrils as binder^29^. The electrode exhibits a high Young’s modulus of approximately 201±12.7 MPa and an electrical conductivity of 95S·cm^−1^; it delivers ~121 mAh·g^−1^ when cycled at C/10 versus a self-standing CF electrode. In another study, an LFP free-standing flexible composite electrode was prepared by the vacuum filtration method with 2D graphene and nanofibrillated cellulose additives^30^. Interestingly, the paper-making process also allowed the electrodes (cathode and anode) and the separator to be integrated into a single flexible and strong structure resulting from successive filtration steps^31^. Numerous examples of cathodes (LFP^29,32^), anodes (MoS_2_^33^, graphite^34^, Si^35^), and even full Li-ion cells (LFP/graphite^31^, LFP/LTO^36^) have been successfully prepared through the paper-making process using different forms of cellulose as binder. Finally, several works have reported flexible electrodes for batteries and supercapacitors that employ cellulose as substrate^37–39^ or binder^40,41^. Most fabrication methods, however, still involve the use of organic solvents, synthetic binders, or high-cost materials that are not easily disposed.

In this paper, a green, inexpensive, rapid, and innovative process for fabricating self-standing Li-ion electrodes is presented. This proposed method particularly avoids the use of resistive and costly binders (i.e., PVDF) as well as volatile and toxic solvents (e.g., NMP). Based on the paper-making process, the method includes the filtration of an aqueous solution that contains millimetric cellulose fibers (FB) with carbon or a mixture of different carbons and redox active materials (LFP, PTCDA, graphite, or LTO). The preparation time of the electrode is considerably reduced because the grinding, component mixing (conductive additive, binder, anode/cathode material), and slurry preparation steps are replaced by a single step to produce a self-standing film. The present method also allows the amount of carbon in the film to be increased because aluminum and copper current collectors are not used. The first steps that is performed is the deposition of cellulose and carbon fibers to form an integrated carbon-rich current collector. The second step involves the formation of the cathode or anode film on the freshly deposited carbon layer. Following this method, both conventional binders and current collectors are not utilized, thus considerably reducing the fabrication cost of the battery. Moreover, substituting the mass of Al/Cu foils with carbon and cellulose fibers results in stronger electrodes and a more conductive film, thereby leading to better performances. This process could also be less expensive than the casting method, which is commonly used in the fabrication of Li-ion electrodes, because low-cost and abundant materials are used (water, PTCDA, and raw cellulose fibers). Tables SI1 and SI2 in the Supporting Information report the price of raw materials used for making electrodes and a cost estimation of both the conventional and the new fabrication method to clearly show the cost saving of our method. In order to facilitate the dispersion of carbons (including graphite as active material) and LFP in water, water-soluble groups, such as aryl-COOH and aryl-SO_3_H moieties, are attached to their surface by diazonium chemistry. Generally, self-standing electrodes reported in literature do not employ such modified carbons or modified LFP, making the proposed process innovative and unique. In this process, commercially available cellulose fibers are utilized with no additional transformations to obtain shorter fibers or modified Z-potentials. In most cases, such as in the paper-making industry, aluminum sulfate hydrate is used as a flocculating agent before carbon is added^32^. This adjuvant is utilized to neutralize the negative charge present in the cellulose fibers by aluminum cations^42,43^. This compound, however, can further react inside the battery; hence, it is removed from our fabrication process. In recent works, it has been demonstrated that microfibrillated cellulose and highly refined cellulose fibers dispersed in water can be effectively used as binder to elaborate self-standing negative electrodes with excellent electrochemical and mechanical performances^34,44^. To obtain fibers in the diameter range of 5–250 nm, acid hydrolysis, and chemical and enzymatic treatments are necessary. These steps, however, incur additional costs and increase preparation time^45^.

In this work, self-standing, flexible, and resistant LFP, PTCDA, graphite, and LTO electrodes are prepared according to a new electrode fabrication method. The electrochemical performances of cathodes and anodes are examined. Long cycling experiments of full LFP/LTO and LFP/graphite batteries reveal that these cells have a remarkably good stability with high Coulombic efficiencies. An all-organic PTCDA/graphite paper battery without a transition metal is prepared and electrochemically tested. This is one of the first self-standing batteries that is composed of organic redox molecules and biodegradable components reported in literature. In the perspective of disposable device production, it is interesting to note that at the end of battery life, the electrodes can be recycled as common paper sheets.

## Experimental Section

### Preparation of cellulose fibers

In the proposed procedure, untreated cellulose fibers (FB) are used to reduce fabrication cost. The sample from Södra black R pulp is used as received. The pulp, which is composed of 2.05–2.25-mm fibers that can be easily beaten, affords exceptionally good binding properties.

For the preparation of a water solution containing cellulose fibers, 400 mg of raw pulp is typically dispersed in 200 mL of deionized water and intensely mixed with an ULTRA-TURRAX disperser for 15 min. The mixture is then cooled to room temperature, and an additional 200 mL of deionized water is added to obtain a 1-g FB/L solution. This aqueous solution is utilized to disperse carbons and active materials.

### Modification of LFP and carbon powders

#### Synthesis of water-soluble carbons

A one-pot technique is utilized to graft aryl-COOH and aryl-SO_3_H moieties on the carbon surface. After the *in situ* formation of diazonium ions, their decomposition by spontaneous reaction with sp^2^ carbon results in a strong carbon–carbon covalent bond^46,47^. Different carbons are chosen and modified with different morphologies. In order to reinforce the mechanical strength of the self-standing film, VGCF carbon fibers with a fiber length of ~10–20 µm are employed to form a carbon current collector with cellulose fibers. Carbon nanotubes (CNT) and spherical Denka carbon are also modified. These two types of carbon are employed to form the cathode and anode on the as-prepared current collector. Graphite is also modified but is only used as an active material.

**Scheme 1:**
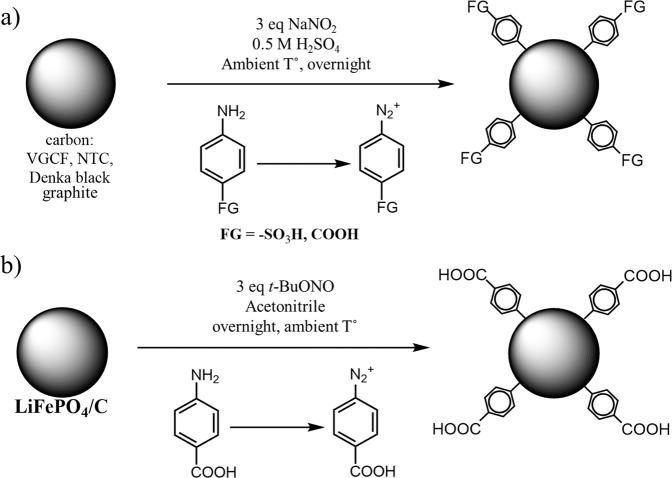
Schematic and conditions of surface modification of a) carbon and b) LiFePO_4_/C powders.

For the modification process, 5 g of carbon is typically dispersed in a 200-mL 0.5-M H_2_SO_4_ aqueous solution. A quantity of *p*-substituted aromatic amine with SO_3_H or COOH groups corresponding to a 0.01 equivalent compared to carbon is then added to the mixture. The mixture is vigorously stirred until the amine is completely dissolved. Thereafter, a 0.03 equivalent of sodium nitrite compared to carbon is added to the mixture to generate the corresponding aryl diazonium ions (N_2_^+^-aryl-SO_3_H or N_2_^+^-aryl-COOH) *in situ*. The mixture is allowed to react overnight at room temperature. Scheme 1 (reaction a) represents the carbon surface functionalization. After the reaction is completed, the mixture is filtered, and the powder is washed several times as described below.

#### Surface modification of LFP powder

LiFePO_4_/C (2–3 wt.% carbon) powder (LFP) is provided by Hydro-Québec, Varennes, Canada. The protocol for the surface modification of LFP with aryl-COOH groups is similar to that for carbon. The reaction, however, is performed in acetonitrile because the LFP slowly reacts with water and is unstable in an acidic medium^48–50^. Sodium nitrite is replaced with tert-butyl nitrite (*t*-BuONO), as shown in Scheme 1 (reaction b).

After an overnight reaction, the mixture is vacuum-filtered using a Büchner assembly and a nylon filter with a 0.22-µm pore size. The modified powder is successively washed with DMF and acetone. Modified carbon powders are washed with deionized water until a neutral pH is reached, followed by DMF and acetone washing. Finally, the modified powders are vacuum-dried at 100 °C for at least one day before utilization.

#### Nomenclature for identifying modified powders

To easily identify the grafted powder, a simple nomenclature is adopted. When aryl-COOH groups are attached to the surface of LFP, Denka, CNT, and VGCF carbons, the powders are named LFP–COOH, Denka–COOH, CNT–COOH, and VGCF–COOH, respectively. When aryl-SO_3_H moieties are immobilized on the carbon surface, the modified powders are identified as graphite–SO_3_H, Denka–SO_3_H, CNT–SO_3_H, and VGCF–SO_3_H.

### Fabrication of self-standing films

**Scheme 2:**
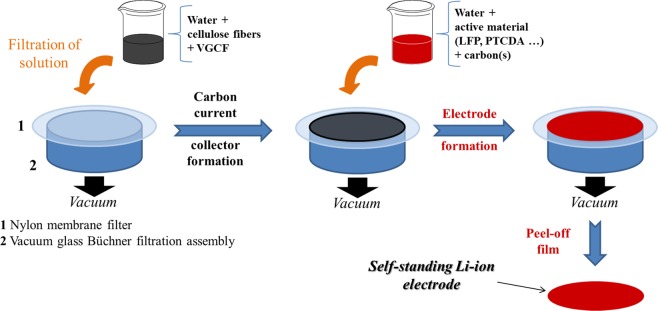
Schematic of new method developed for the fabrication of self-standing electrode films. The first step is the formation of a carbon current collector made of cellulose fibers and VGCF. The second step is the formation of a Li-ion electrode directly on the carbon current collector (red color refers to PTCDA cathode material).

**Figure 1:**
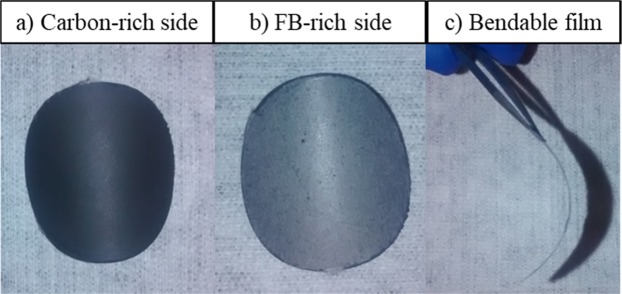
Pictures of carbon current collector composed of cellulose fibers and VGCF. (**a**) The carbon-rich side is obtained to collect electrons and (**b**) the FB-rich side is essential to trap the active material and carbon during the second filtration step. (**c**) The film is completely bendable.

**Figure 2:**
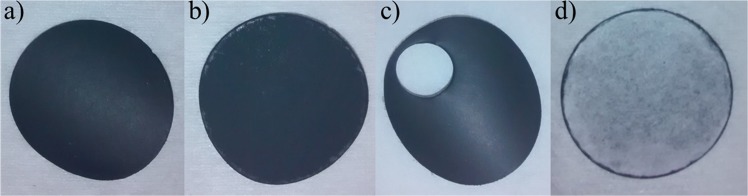
Images of a self-standing LFP electrode. (**a**) The carbon-rich side remains shiny, whereas (**b**) the active material (LFP) fills the pores of the paper matrix. (**c**) After cutting a small electrode, the film remains intact and resistant. (**d**) Self-standing film with a carbon current collector, an integrated LFP electrode, and a paper separator.

Scheme 2 shows the new method of Li-ion electrode fabrication. An adequate volume of the aqueous solution that contains cellulose fibers (1 g FB/L) is placed in a beaker; typically, a volume that corresponds to 30 mg of FB is employed. Thereafter, 10 mg of VGCF, VGCF–SO_3_H, or VGCF–COOH is dispersed into the solution. They are all stirred using an ultrasonic rod. The modified VGCF powders are instantly solubilized in water, whereas at least 10 min is necessary to disperse the commercial VGCF. After a homogeneous solution is obtained, the content is vacuum-filtered on a 47-mm diameter size nylon membrane with a 0.22-μm pore to form the carbon current collector; a typical carbon current collector is shown in Fig. 1. The film where a mass of electrode active material (LFP, PTCDA, graphite, or LTO) in the range 40–70 mg is dispersed in deionized water is allowed to dry for 10 min. This is followed by the addition of 10 mg of carbon, which could be modified or unmodified VGCF, Denka, CNT, or a mixture of these three. In all cases, the modified LFP and carbons are easily and more rapidly dispersed in water, thus leading to a better film appearance. After mixing, the solution is gently poured on the as-prepared carbon current collector to form the electrode. The film is peeled off the filter to obtain the self-standing Li-ion electrode. The electrode is thereafter calendered at room temperature, i.e., 50 or 80 °C. The produced film is placed in a vacuum oven and heated at 130 °C for at least 1 day before it is utilized in the battery. Figure 2 shows an LFP electrode made according to the new fabrication method.

As an option, a third step can be included in order to directly create a paper separator on the electrode film (Fig. 2b). An aqueous solution, which contains 30 mg of FB, for instance, can be filtered on the fresh electrode. Figure 2d shows a self-standing film composed of a carbon current collector, an integrated LFP electrode, and a paper separator.

### Characterizations

Prior to the analysis, the samples are vacuum-dried at 120 °C for several hours. The self-standing electrodes are observed with a FlexSEM 1000 scanning electron microscope (Hitachi High-Technologies Corporation) in a dry room. Secondary electron images are obtained at an accelerating voltage of 5 kV and a working distance of approximately 5–5.5 mm with various magnifications. Three-dimensional confocal microscopy images and corresponding optical images are obtained with a Keyence VK-x200 3D laser microscope at 150× magnification.

### Cycling procedures

#### Cell assembly

To individually test the various electrodes prepared, two-electrode electrochemical coin cells are assembled with a lithium metal counter and a reference electrode. Celgard-3501 polymer or Kodoshi paper is used as separator and soaked with a 1-M LiPF_6_ EC:DEC (3:7) electrolyte. For a complete battery, the LFP, PTCDA, graphite, and LTO self-standing films are assembled together with a Celgard or Kodoshi paper separator. A bare electrode is also tested using a stainless steel spacer as a working electrode. In order to verify the electrochemical inertness of cellulose fibers, a handmade paper electrode is tested versus lithium.

The cells are assembled in an argon-filled glove box with an oxygen level that is less than 10 ppm and thereafter controlled with a VMP3 potentiostat.

#### Electrochemical testing

Cyclic voltammetry. The electrochemical behaviors of bare electrode (stainless steel spacer only), paper electrode (composed only of FB), and self-standing electrode films made of LFP, PTCDA, and LTO are compared through cyclic voltammetry experiments. A scan rate of 0.03 mV·s^−1^ is employed among the various potential windows.

For bare and paper electrodes, the experiment starts from an open circuit potential (OCP) to 4.2 V. It is then followed by a reverse scan of 2.0 V vs. Li/Li^+^. The same procedure is followed for the LFP electrodes except that the upper cutoff voltage is set to 4 V. For the LTO electrodes, the experiment starts from the OCP to 1.2 V. It is thereafter followed by a reverse scan of 2.5 V vs. Li/Li^+^. For the PTCDA electrodes, the experiment starts from the OCP to 1.5 V followed by a reverse scan of 3.5 V vs. Li/Li^+^.

Galvanostatic cycling and long cycling experiments. The charge/discharge cycling procedure is performed in a galvanostatic mode at different current density ranges of 2.0–4.0 V, 1.2–2.5 V, 1.5–3.5 V, and 0–1.5 V versus Li/Li^+^ for the LFP, LTO, PTCDA, and graphite electrodes, respectively. For each cycling rate ranging from C/10 to 5 C, five cycles are subsequently recorded. The experiments are automatically started with two formation cycles at C/24.

Additionally, LFP/LTO cells with mass ratios of 1 and ~0.85 are also tested between 1.0 and 2.5 V versus the LTO at different cycling rates ranging from C/24 to 5 C.

Long cycling experiments at C/10, C/2, 1 C, or 2 C rates are also recorded after several cycles of formation at different C rates for PTCDA/Li, graphite/Li, LTO/Li, LFP/LTO, LFP/graphite, and PTCDA/graphite batteries. The prelithiation of the PTCDA cathode is performed in a coin cell with a lithium anode by discharging at C/24 from the OCP to 1.5 V versus Li/Li^+^. It is followed by a chronoamperometry experiment where a constant potential of 1.5 V is maintained for 3 h. The coin cell is thereafter disassembled, and the prelithiated PTCDA cathode is recovered to be assembled with a graphite anode.

All electrodes are cut into small circular discs (area = 1.13 cm^2^; Fig. 2c) with a mass loading range of 8.4–11.2 mg·cm^−2^ depending on the quantity of active materials inserted inside the film.

## Results and Discussion

### Fabrication of self-standing films

As shown in Figs. 1 and 2, thin films that are approximately 100-µm thick are obtained through the aforementioned fabrication technique. The calendering of self-standing films results in reduced thickness, as discussed below. Generally, average thickness reductions of 20 and 30% are observed when the electrodes are calendered at 50 and 80 °C, respectively. The films are fully bendable, rollable, and resistant, as shown in Fig. 1. Punching an electrode from the film does not affect the integrity of the electrode, as shown in Fig. 2c. The carbon-rich side produced during the first fabrication step (Fig. 2a) remains totally similar after the filtration of the solution that contains the active material. The FB-rich side shown in Fig. 1b is then filled with active material and carbon during the second filtration step. A two-layer electrode film composed of a carbon current collector, a layer of active material, and carbon is subsequently fabricated. This method is interesting because it avoids the inclusion of the mass of an aluminum current collector. This mass can be replaced with more cellulose fibers or carbon in order to fabricate a strong film with a higher carbon content than a film casted on a metal current collector. In fact, for an electrode (~6 mg/cm^2^ of active material) casted on an aluminum foil, the active material (e.g., LFP) accounts for 53% of the total mass, whereas the inactive aluminum is 41 wt.%. Approximately 6% of the electrode mass corresponds to the binder and carbon. Another interesting aspect is that the quantity of active material may be accurately determined because it is weighed before it is introduced to the beaker and before the filtration step (Scheme 2). Depending on the battery configuration or cathode/anode material utilized, the quantity of active materials (in mg·cm^−2^) in the self-standing film can be easily adapted.

**Figure 3:**
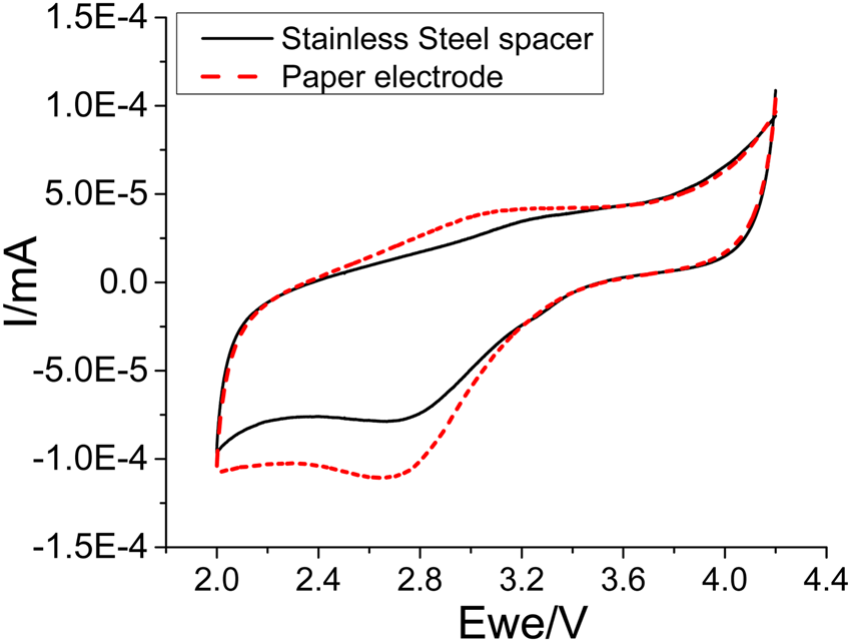
Cyclic voltammetry experiments performed at a scan rate of 0.03 mV.s^−1^ for bare electrode (stainless steel spacer only) (—) and paper electrode composed of FB only) ().

**Figure 4:**
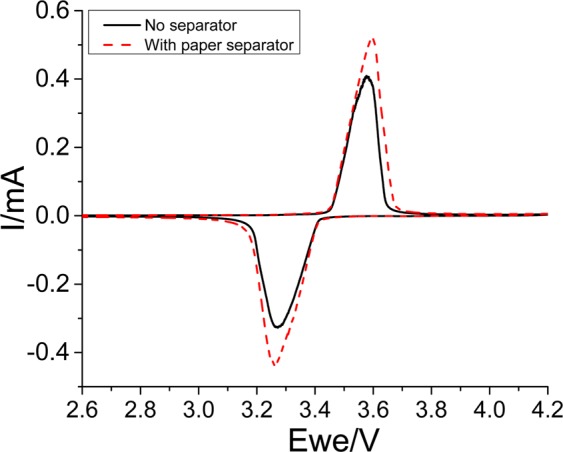
Cyclic voltammetry experiments performed at a scan rate of 0.03 mV·s^−1^ for a self-standing LFP electrode with () and without (—) an integrated paper separator deposited on top of the electrode.

In order to confirm that raw cellulose fibers can be used as a support for the active material in a Li-ion battery, a simple paper electrode entirely composed of cellulose fibers is fabricated. This electrode is tested as a working electrode in a coin cell versus lithium metal. The cycling voltammetry experiment shown in Fig. 3 reveals that the cellulose fibers () are totally electro-inactive up to 4.2 V, thus confirming their possible integration in Li-ion battery manufacturing. In fact, the electrochemical response in the potential window from 2 to 4.2 V vs. Li/Li^+^ is similar to that obtained in a bare electrode (**—**) that is only composed of a stainless steel spacer. The currents recorded are approximately 10^4^ lower than those obtained when an LFP cathode is cycled, as shown in Fig. 4.

In the new fabrication process, a third step can be included. As an option, a paper separator can be directly deposited onto the electrode film, as shown in Fig. 2d. Cyclic voltammetry is employed to characterize the produced film and verify the influence of the paper separator on the electrochemical properties. A Celgard separator is employed to compare the electrochemical behavior of an electrode without an integrated paper separator. Figure 4 shows the cyclic voltammetry experiments performed at a scan rate of 0.03 mV·s^−1^ on a self-standing LFP electrode with (—) and without () a paper separator added on top of the electrode. The obtained results indicate that the additional layer does not adversely affect the electrochemical properties of the LFP electrode because the polarizations (∆E) of the two electrodes are similar. Furthermore, the intensity of redox peaks of the electrode integrated with the paper separator is slightly higher than that of the LFP electrode alone. This can be explained by the enhancement of mechanical properties and the increased integrity of the film. A similar behavior resulting from the use of water-soluble carbons is observed. These water-soluble carbons yield a stronger film and lead to a better penetration of carbon and active material into the voids created by the cellulose matrix.

### Morphological analyses of self-standing films

**Figure 5:**
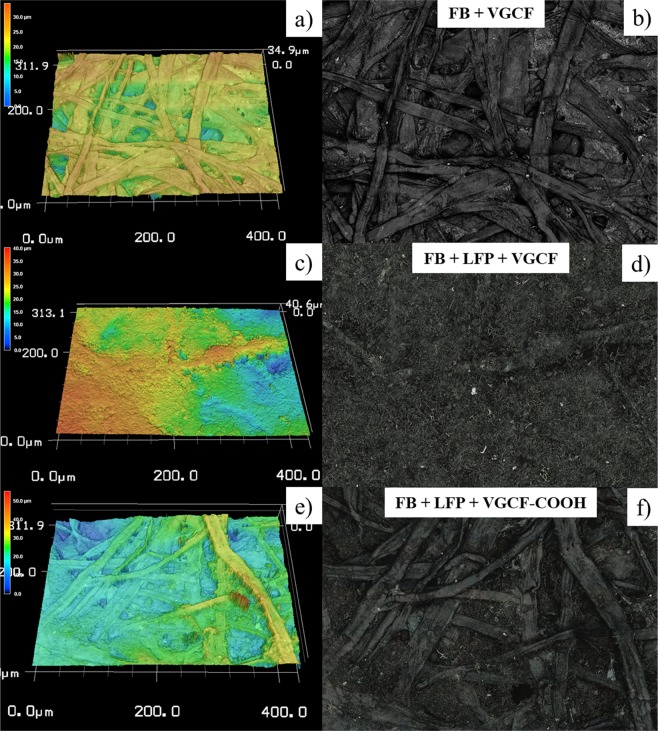
3D confocal microscopy images and corresponding optical images of cellulose films made of the following: (**a,b**) VGCF carbon; (**c,d**) VGCF carbon and LFP; (**e,f**) VGCF–COOH carbon and LFP.

The surface topology of cellulose electrode films made of unmodified and modified VGCF carbons and with/without LFP is obtained through 3D confocal microscopy experiments. Figure 5 shows the optical and 3D confocal microscopy images of the FB-rich side of these different cellulose films. In the first fabrication step, the carbon current collector is created, thus producing carbon-rich and FB-rich sides, as shown in Fig. 1. The FB-rich side is particularly inhomogeneous and highly porous, as shown in Fig. 5a,b. After the second step, which involves the filtration of the mixture of LFP and unmodified VGCF carbon, the surface appears smoother (Fig. 5c,d) and is mainly composed of carbon that has not migrated inside the electrode. The poor dispersion of carbon in the electrode results in an inferior electrochemical performance, which is worse than the performance of an electrode made of modified VGCF-COOH carbon. In fact, after aryl-COOH groups are grafted, the carbon fibers become more soluble in water and are well-distributed in the bulk of the electrode. The FB-rich side of the LFP electrode made of VGCF–COOH (Fig. 5e,f) is basically similar with that obtained for the cellulose film without an active material (Fig. 5a,b) except that some voids are filled with LFP and carbon. This is attributed to the better penetration of modified carbon into the pores of the electrode.

**Figure 6:**
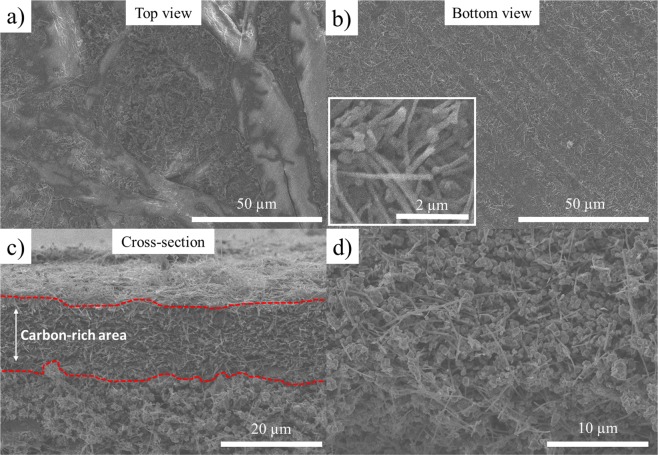
(**a**) Top view, (**b**) bottom view, and (**c,d**) cross-section SEM images of an LTO self-standing film made of VGCF–COOH carbon obtained at magnifications of (**a,b**) ×1000, (**c**) ×3000, and (**d**) ×4000. The inset in (**b**) is a zoom-in image showing the intertwined carbon fibers. The red dots in (**c**) identify the carbon current collector.

**Figure 7:**
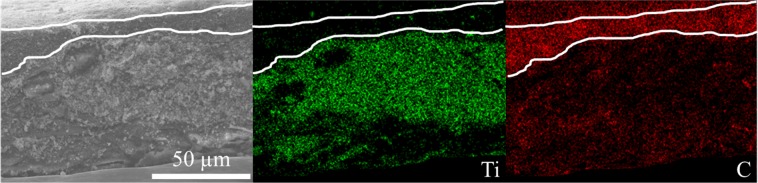
Cross-section SEM image of an LTO self-standing film made of VGCF–COOH carbon and corresponding elemental mapping of Ti (green) and C (red). White lines identify the carbon current collector.

The SEM images of an LTO self-standing film are presented in Fig. 6. The top (Fig. 6a) and bottom views (Fig. 6b) represent the FB-rich and carbon-rich sides, respectively. As shown in the top-view image, the pores created among the large cellulose fibers is filled with the mixture of modified VGCF and LTO materials. The corresponding C and Ti elemental mappings are shown in Fig. SI1, indicating that carbon and LTO exhibit good dispersion. The bottom view (Fig. 6b) only shows the VGCF–COOH fibers that form a strong carbon mat (which is expected) that is ideal for electron conduction and current collection. The inset shows the intertwined carbon fibers that reinforce the electrode film. Finally, the cross-section of the electrode film presented in Fig. 6c shows a carbon-rich area that is approximately 20 µm thick with no (or extremely few) LTO particles. Inside the electrode (Fig. 6d), a composite of well-dispersed LTO particles with interconnected VGCF carbon fibers is observed; this composite permits strong electrode adhesion and good electrical conduction. The chemical mapping of the cross-section of this self-standing film that illustrates Ti and C dispersions inside the electrode is shown in Fig. 7. A high carbon concentration is effectively located on one side of the self-supported film with the presence of an extremely small amount of titanium. The LTO material is well-dispersed in the bulk of the film, demonstrating that thick film electrodes with high active mass loading can be easily obtained with this technique. Carbon is also well-dispersed, ensuring good electrical conduction throughout the film thickness, as demonstrated by the electrochemical performance of self-standing electrodes presented below.

### Electrochemical performance of LFP electrodes

**Figure 8:**
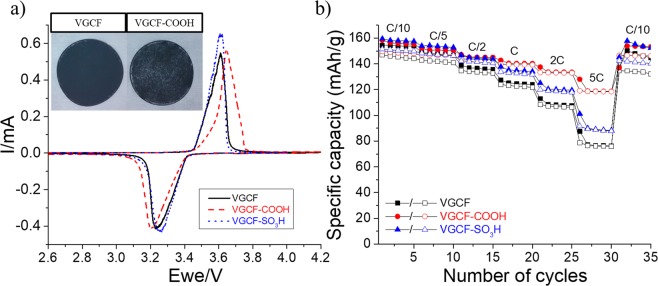
(**a**) Cyclic voltammetry experiments performed at a scan rate of 0.03 mV·s^−1^ for three LFP electrodes containing VGCF (—), VGCF–COOH (), and VGCF–SO_3_H (· · · ·) as carbon additives. The inset shows a photograph of self-standing films made of VGCF and VGCF–COOH carbons. (**b**) Comparison of rate capabilities among the same electrodes. Full and empty characters represent charges and discharges, respectively.

Figure 8 shows the (a) cycling voltammetry and (b) rate capability experiments performed for the three LFP electrodes with the same loading and conducted according to the same procedure. Three different carbons, however, are used: unmodified VGCF (—), COOH-aryl (), and SO_3_H-aryl-modified VGCF (· · · ·). Although the cyclic voltammograms are relatively similar, Fig. 8b shows a clear improvement of the electrochemical performance when modified VGCFs are used, especially at 2 C and 5 C cycling rates. At a low current density (C/10), the discharge capacities of all electrodes are similar, i.e., approximately 155 mAh·g^−1^. When the cycling rate is increased to 1 C, the discharge capacity remains at approximately 140 mAh·g^−1^ for electrodes that contain the modified VGCF. On the other hand, for the film made of VGCF, 120 mAh·g^−1^ is obtained. At a 5 C rate, a similar discharge capacity of 120 mAh·g^−1^ is obtained for the electrode containing the COOH-aryl-modified VGCF. As discussed above, water-soluble carbons are well-dispersed in the pores created by cellulose fibers, thus resulting in better electrical conductivity in the film. At 5 C, approximately 50 and 77% of the initial discharge capacity is recovered when the VGCF and COOH–aryl-modified VGCF are employed, respectively. The inset in Fig. 8a shows that cellulose fibers are visible on the surface of the electrode made of VGCF–COOH carbon. This observation confirms that the modified carbon migrates inside the film during the process, thus leading to a better contact between the LTO and carbon fibers (Fig. 6d). Similar electrochemical improvements were reported by Wu *et al*. with their cathode composite made of carbon-coated LFP nanoparticles that are electronically connected with CNTs, thus forming an optimal three-dimensional conducting network^51^.

Modified Denka and CNT carbons were also tested in the composition of the electrodes and the electrochemical results of such cathodes are shown in Fig. SI3. Despite water-soluble CNTs gave a small improvement of electrochemical performance at high C-rates, the modified VGCF remains the best choice for making the electrode for economic reasons.

**Figure 9:**
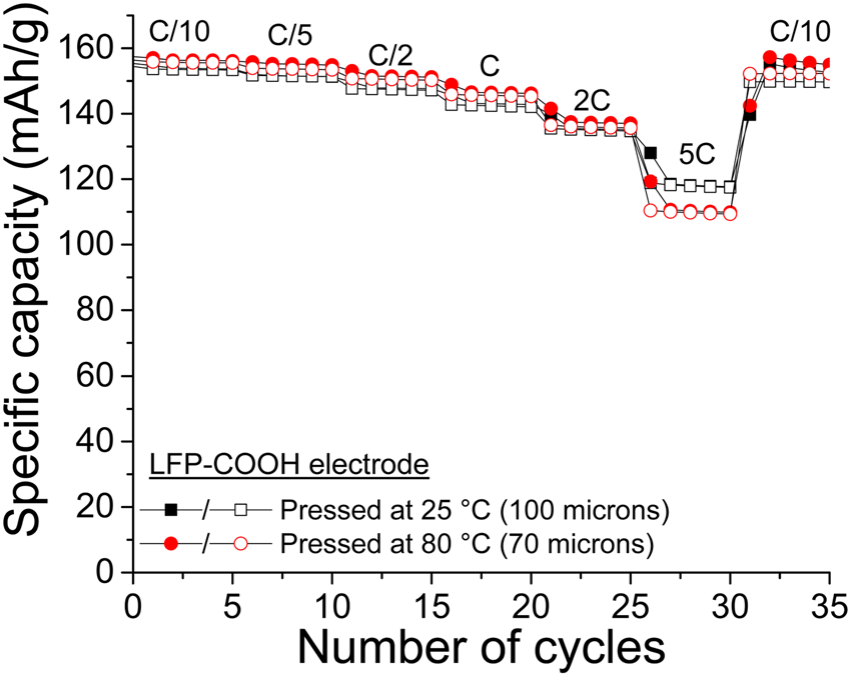
Comparison of rate capabilities of an LFP electrode made of modified LFP–COOH material calendered at 25 (▪) and 80 °C (). Full and empty characters represent charges and discharges, respectively.

The use of water-soluble carbons demonstrates that better electrochemical performance and shorter preparation time can be achieved; accordingly, the modified LFP with aryl-COOH groups is prepared. The same method for carbon modification is employed but with certain differences. The reaction procedure is represented in Scheme 1 (reaction b). The modification is conducted in acetonitrile rather than acidic media because the LFP powder rapidly reacts with water and acids^48–50^. Figure 9 shows the electrochemical performances of two LFP electrodes made of modified LFP–COOH materials with different thicknesses. First, it should be noted that the grafting of organic species on the LFP does not adversely affect the electrochemical performance because the rate capability is practically identical regardless of whether pristine or modified LFP is used. Although this result has not been verified, it indicates that the amount of grafted groups should be extremely low (<1 wt.%) as indicated by the performances remaining unchanged. An extremely high loading of grafted groups leads to an unsatisfactory rate capability, as demonstrated by recent works^47,52^. In conclusion, the use surface-modified carbon and LFP–COOH allows the fabrication of a strong self-standing film in less than 10 min by simply mixing the components in water. As shown in Fig. 9, a slightly higher capacity is obtained after calendering at 80 °C because the contact between the carbon and active material inside the electrode is better. The same result is obtained with the unmodified LFP electrodes calendered at 50 °C (Fig. SI2; Supporting Information). At 25 °C, an approximately 100-µm thick film is obtained, whereas ~80 and ~70 µm are measured after calendering at 50 and 80 °C, respectively.

### Electrochemical performance of LTO electrodes

The LTO (Li_4_Ti_5_O_12_) is an active material that is being considered for application in Li-ion batteries^53^. This material does not form a solid electrolyte interface (SEI) layer because its working voltage occurs at a sufficiently high potential (1.55 V vs. Li/Li^+^). Moreover, the LTO qualifies as a zero-strain material because it remains stable during the insertion/deinsertion of lithium^54^. Similar to the LFP, the LTO is a relatively inexpensive material and therefore a perfect candidate for the production of low-cost batteries.

**Figure 10:**
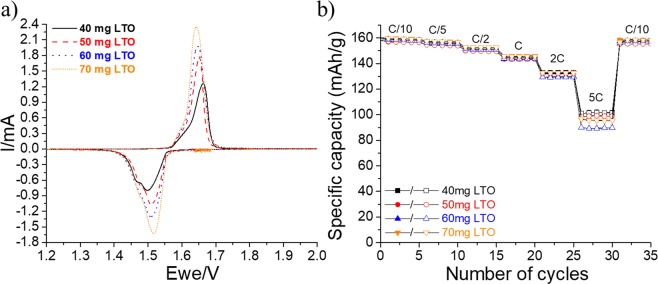
(**a**) Cyclic voltammetry experiments performed at a scan rate of 0.03 mV·s^−1^ for four LTO electrodes with different amounts of active material. (**b**) Comparison of capability rates of the same electrodes. Full and empty characters represent charges and discharges, respectively.

Self-standing LTO films with different loadings are prepared following the same method of fabrication described in Scheme 2. The cyclic voltammetry and rate capability experiments are presented in Fig. 10. When the load is increased (because of the LTO mass), the intensity of redox peaks in the voltammogram (Fig. 10a) increases, and the polarization slightly decreases. This behavior demonstrates that this technique of fabrication can be adapted for high loads of active material. Furthermore, the corresponding rate capabilities shown in Fig. 10b are considerably similar up to a rate of 2 C. At 2 C, approximately 135 mAh·g^−1^ is obtained, representing ~85% of the initial discharge capacity (160 mAh·g^−1^). A few slight variations among the anode capacities are observed at 5 C; nevertheless, approximately 90–100 mAh·g^−1^ is delivered. Similar to the LFP cathodes, a better electrochemical performance is obtained when a small amount of CNT–COOH is added to the composition of the LTO electrode. For instance, at a 5 C rate, a specific capacity of 120 mAh·g^−1^ is obtained (Fig. SI4; Supporting Information).

**Figure 11:**
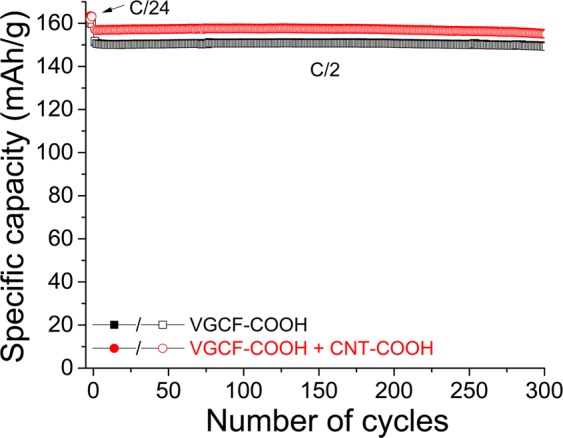
Long cycling experiments performed at a constant charge/discharge current of C/2 between 1.2 and 2.5 V versus Li/Li^+^. Two LTO electrodes, one composed of VGCF–COOH (▪) and the other of a mixture of VGCF–COOH and CNT–COOH (), are compared. Full and empty characters represent charges and discharges, respectively.

The cyclability of LTO self-standing electrodes is evaluated with long cycling experiments at a rate of C/2. Figure 11 presents the capacity retention of two different compositions of electrodes with more than 300 charge/discharge cycles. Practically 100% of the initial discharge capacity at C/2 is recovered at the end of experiment. Stable discharge capacities of 157 and 150 mAh·g^−1^ are thus obtained through several hundreds of cycles when the electrode is fabricated with () and without CNT–COOH (▪). Moreover, a stable Coulombic efficiency of appoximately 99.9% is obtained by both electrodes at 300 cycles. After cycling, the cell is disassembled, and the self-standing electrode remains totally intact without the dissolution of active material or carbon in the electrolyte (Fig. SI5; Supporting Information).

### Electrochemical performance of complete LFP/LTO batteries

Complete Li-ion batteries are assembled with the self-standing LFP and LTO electrodes. The LTO anode material is in the delithiated state and three Li^+^ ions can be inserted according to Eq. (1)^55^.

1 $$3{\rm{L}}{\rm{i}}{\rm{F}}{\rm{e}}{\rm{P}}{{\rm{O}}}_{4}+{\rm{L}}{{\rm{i}}}_{4}{\rm{T}}{{\rm{i}}}_{5}{{\rm{O}}}_{12}\to 3{\rm{F}}{\rm{e}}{\rm{P}}{{\rm{O}}}_{4}+{\rm{L}}{{\rm{i}}}_{7}{\rm{T}}{{\rm{i}}}_{5}{{\rm{O}}}_{12}$$

The practical capacity of 165 mAh·g^−1^ for the LTO electrode is slightly higher than that obtained for the LFP self-standing film (160 mAh·g^−1^); hence, electrochemical cells with an LFP/LTO mass ratio of approximately 1 can be used. Apart from this ratio, however, the use of a minimal amount of excess LTO material achieves an LFP/LTO mass ratio of ~0.85. This excess is preconized for safety concerns and is generally employed in commercial batteries. A full Li-ion battery is assembled with a standard Celgard separator and another with a Kodoshi paper separator to produce an all-paper battery. Compared to the cell assembled with a Celgard separator, the battery that employs a paper separator is observed to achieve a better electrochemical performance: for every C rate ranging from C/24 to 2 C, 10 mAh∙g^−1^ is added to the obtained discharge capacity (Fig. SI6; Supporting Information).

**Figure 12:**
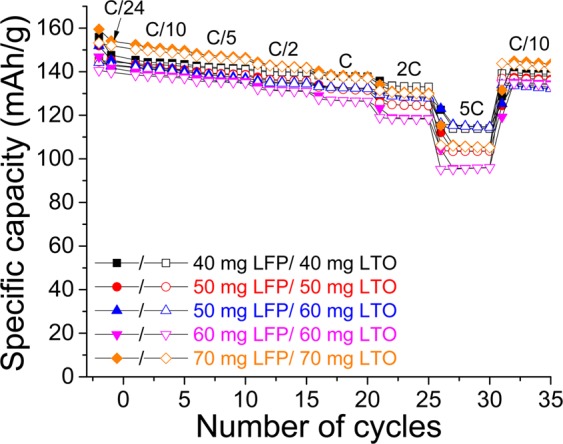
Comparison of rate capabilities of LFP/LTO batteries made with different amounts of LFP and LTO and with LFP/LTO mass ratios of 1 and ~0.85. Paper Kodoshi separator is used for all electrodes. Full and empty characters represent charges and discharges, respectively.

The electrochemical performance is significantly better when a paper separator is employed; hence, several LFP/LTO batteries with different amounts of active materials and Kodoshi paper as separator are assembled. According to the fabrication method described in Scheme 2, LFP and LTO masses in the range 40–70 mg are utilized. Li-ion batteries with LFP/LTO mass ratios of 1 and ~0.85 are electrochemically tested; the rate capabilities of such electrodes are shown in Fig. 12. The results do not exhibit particular differences when an excess amount of anode material is utilized, which is a valid observation. A remarkable improvement, however, is evident when the battery is cycled at a high C rate. In fact, at a 5 C rate, for the two electrodes made of 50 mg of LFP, the specific capacities obtained are approximately 105 and 115 mAh·g^−1^ when LFP/LTO mass ratios of 1 and 0.85 are utilized, respectively. Under these conditions, a better retention capacity at high C rates should therefore be obtained; however, additional results should be obtained to confirm this observation. Overall, the specific capacities obtained are slightly lower when the anode and cathode loadings are increased especially at high C rates. For instance, a battery that is assembled with electrode films made of 40 mg of LFP and LTO (▪) delivers a capacity of 135 mAh·g^−1^ at 2 C. On the other hand, 125 mAh·g^−1^ () and less than 120 mAh·g^−1^ () are obtained when films composed of 50 and 60 mg of LFP and LTO are employed, respectively. The same behavior is observed at 5 C because 115, 105, and 95 mAh·g^−1^ are delivered by the electrode films fabricated with 40, 50, and 60 mg of active materials, respectively.

**Figure 13:**
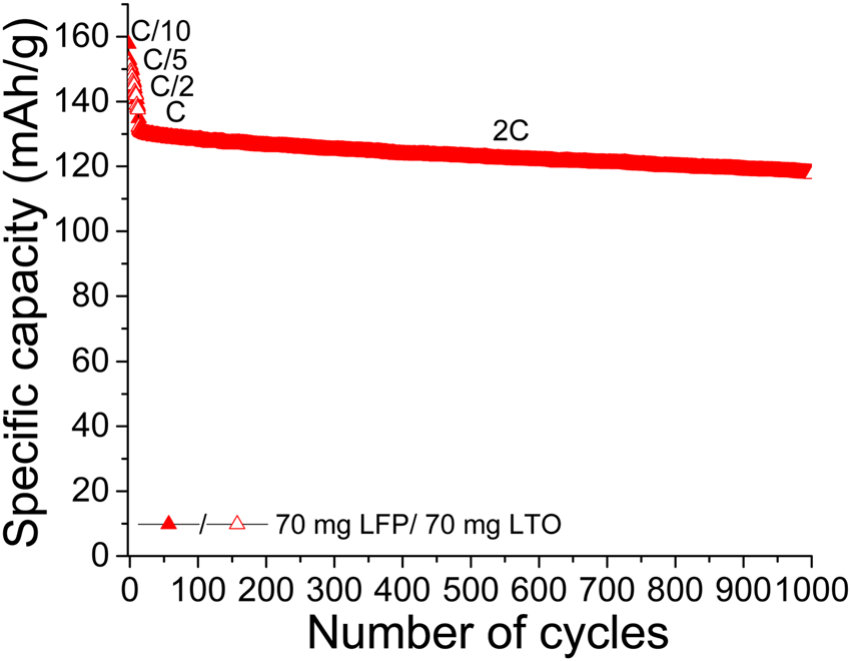
Long cycling experiment conducted at a constant charge/discharge current of 2 C between 1.0 and 2.5 V versus LTO for an LFP/LTO battery made of 70 mg LFP and LTO; a paper Kodoshi separator is used. Full and empty characters represent charges and discharges, respectively.

The cyclability of a complete LFP/LTO battery made of 70 mg of active material is evaluated over 1000 cycles at a 2 C rate after several cycles of formation (Fig. 13). An initial discharge capacity of approximately 130 mAh·g^−1^ is obtained with a high capacity retention of ~91.6% after 1000 cycles. To the best of our knowledge, the foregoing is one of the best results reported in literature for an all-paper full cell. In contrast, the LFP/LTO battery reported by Cornell *et al*.^36^ delivered an initial discharge capacity of 140 mAh·g^−1^ at C/10. Apart from a low coulombic efficiency, a poor capacity retention of 87% is observed after only 20 cycles. The decrease in the active mass loading is found to deteriorate the cyclability of the battery. In fact, the same experiment with electrodes made of 40 mg of LFP and LTO has resulted in a capacity retention of only 82.9% (Fig. SI7; Supporting Information). In both cases, however, a high Coulombic efficiency of ~99.9% is obtained. This observation may possibly be explained by the better contact between the active material (LFP or LTO) and carbon in the heavier film, leading to slightly higher capacities and avoiding the progressive electrical isolation of the active material (i.e., better capacity retention). This capacity loss is already observed among electrodes made of LFP sub-micrometer plates^56^ and micro-sized LFP^57^ cycled at 1 C. Further cycling experiments at C/2 among different electrode compositions are performed, and the same conclusion is obtained. These results are shown in Fig. SI8b, which also shows that capacity retentions of 89.6, 90.8, and 91.4% are obtained when 40, 60, and 70 mg of LFP are used, respectively.

### Electrochemical performance of PTCDA electrodes

Organic electrodes are known to be potential candidates for next-generation Li-ion batteries^9,58^. Organic electrodes could lower the cost of battery manufacturing because organic materials can be prepared from natural products and biomass^59^. Moreover, because of the absence of inorganic structures and metals, such as cobalt or nickel, batteries made of organic materials are eco-friendly and totally recyclable. The possible fabrication of a self-standing organic cathode film that is solely composed of recyclable materials (cellulose, carbon, and redox-active organic molecule) is therefore demonstrated. A number of organic molecules have been reported as possible active materials in Li-ion batteries^60–63^. For our experiments, an inexpensive commercial 3,4,9,10-perylenetetracarboxylic dianhydride (PTCDA) molecule, which has a theoretical capacity of 273 mAh·g^−1^, is selected.

**Figure 14:**
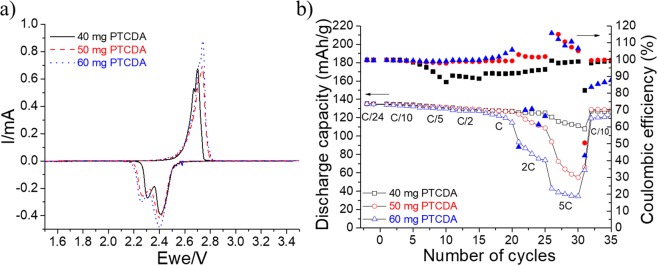
(**a**) Cyclic voltammetry experiments performed at a scan rate of 0.03 mV·s^−1^ for three PTCDA electrodes containing a mixture of VGCF–SO_3_H and CNT–SO_3_H. (**b**) Comparison of rate capabilities for the same electrodes. Full and empty characters represent the Coulombic efficiency (%) and discharge capacity, respectively.

Self-standing PTCDA cathodes with different active mass loadings are prepared according to the method described in Scheme 2. This organic molecule is partially soluble in water; hence, the preparation of cathode is considerably simple, and the PTCDA is securedly contained in the cellulose/VGCF substrate. The cyclic voltammetry and rate capability experiments for the PTCDA electrodes are presented in Fig. 14. This organic molecule is known to be a semiconductor^64^, and its rate performance and stability are considerably poor because of its low electrical conductivity and high solubility in an electrolyte. In fact, the increase in the PTCDA mass in the self-standing film has also increased polarization (Fig. 14a) although the intensity of the redox peak has slightly increased. This behavior is confirmed by the rate capability experiments shown in Fig. 14b. During the discharge of PTCDA electrodes, the voltage suddenly drops, as reported by recent articles on organic cathode materials^65,66^. The initial drop from the OCP to a plateau at approximately 2.4 V vs. Li/Li^+^ is caused by the transformation from the PTCDA to lithium enolate with the assimilation of Li^+^ ions. The specific capacity obtained, however, is approximately 137 mAh·g^−1^, which corresponds to only half of the theoretical capacity because only two lithium ions react with ketones^67^. The insertion of the other two Li^+^ ions occurs in the potential window range of 0.9–1.3 V vs. Li/Li^+^. An intense discharge process, however, will damage the structure and therefore result in a strong irreversibility^65^. When self-standing PTCDA electrodes are cycled at C rates that exceed 1 C (Fig. 14b), the discharge capacities considerably decrease with the increase in active mass loading. The discharge capacities are therefore approximately 110, 60, and only 35 mAh.g^−1^ at 5 C for PTCDA electrode films composed of 40, 50, and 60 mg of active materials, respectively. These electrochemical performances, however, are better than those generally reported for electrodes spread on aluminum current collectors with inferior active mass loadings^67^. As an example, following the new fabrication method, an approximately 6-mg·cm^−2^ PTCDA electrode is capable of delivering 120 mAh·g^−1^ at 1 C.

**Figure 15:**
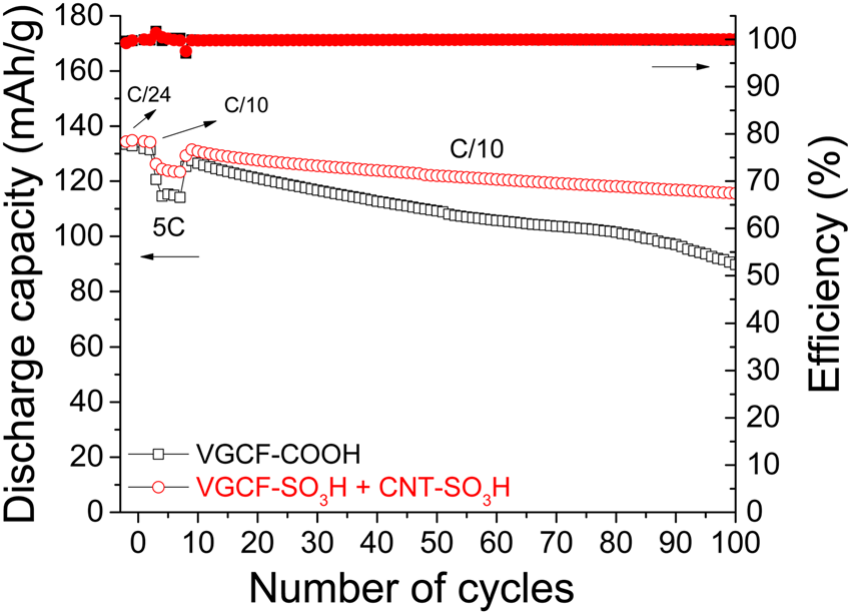
Long cycling experiments performed at a constant charge/discharge current of C/10 between 1.5 and 3.5 V versus Li/Li^+^. Two PTCDA electrodes (40 mg), one composed of VGCF–COOH (□), and the other of a mixture of VGCF–SO_3_H and CNT–SO_3_H (), are compared. Full and empty characters represent the Coulombic efficiency (%) and discharge capacity, respectively. Before the long cycling experiment, a formation rapidly occurs at 5 C.

Figure 15 presents the capacity retention over 100 charge/discharge cycles for two electrodes with different compositions after performing a rapid formation at 5 C. This formation has prevented the migration of PTCDA in the electrolyte, and the Coulombic efficiency has remained at approximately 100% in all the cycling experiments. Electrochemical performance for battery doublons cycled without the formation at high C-rate are presented in Fig. SI9. In this case, a rapid decrease of Coulombic efficiency was observed with cycling. Post-mortem microscopy analyses of the cathode surface after cycling at a 5 C rate should be performed to understand the reasons that cause the better electrochemical performance. It is probable that during cycling at a high C rate, the PTCDA molecules do not have sufficient time to migrate to the electrolyte and are confined in the cathode. During the same time, a passivation film is formed at the surface of the cathode because of electrolyte degradation. This layer could impede the PTCDA dissolution in the electrolyte without affecting the electrochemical performance.

Although the resulting Coulombic efficiency becomes better by rapidly performing five cycles at 5 C, this improvement has no significant impact on the cyclability because 80 and 91% of the initial discharge capacity at C/10 are obtained after 70 charge/discharge cycles at C/10 for the electrodes composed of VGCF–COOH (Fig. 15, □) and a mixture of VGCF–SO_3_H and CNT–SO_3_H (Fig. 15, ), respectively. These values are relatively similar to those calculated in the galvanostatic cycling experiments shown in Fig. SI9. It is evident that the use of a small quantity of CNT–SO_3_H in the composition of the electrode enhances not only the discharge capacity delivered but also the cyclability over hundreds of cycles. The rapid capacity loss observed after 80 charge/discharge cycles in the self-standing film composed of VGCF–COOH is probably caused by the degradation of the Li metal anode^68^. This is confirmed by the additional electrochemical results obtained using PTCDA electrodes with higher active mass loadings (Fig. SI10, Supporting Information), which sustain a rapid capacity decay after only 70 cycles. In fact, the increase in active mass induces the increase in the cycling current, which promotes the formation of lithium with a high surface area and leads to an insufficient cycle life^69^.

The method of fabrication described in this work is clearly adapted to the use organic cathode materials. The electrochemical performance (capacity and Coulombic efficiency) obtained is considerably better than those usually reported for PTCDA cathodes with lower active mass loadings^70^. Moreover, to avoid the shuttle effect on the PTCDA electrode, the rapid formation at 5 C is considered and should be applied to other high-capacity organic molecules, such as anthraquinone^71^ or 1,4,5,8-naphthalenetetracarboxylic dianhydride^72^.

### Electrochemical performance of complete LFP/graphite batteries

**Figure 16:**
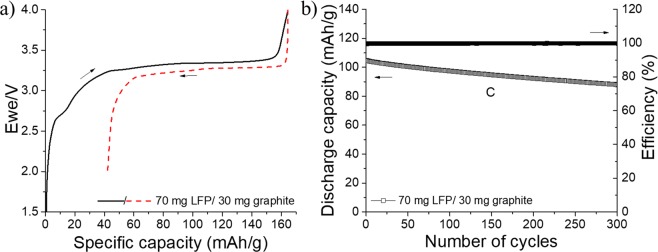
(**a**) First charge (—) and discharge () obtained at a constant current of C/24 between 2 and 4 V versus graphite in an LFP/graphite battery. A paper Kodoshi separator is used, and an excess of the anode capacity is employed. (**b**) The corresponding long cycling experiment is performed at a 1 C rate. The full and empty characters represent the Coulombic efficiency (%) and discharge capacity, respectively.

In order to produce a low-cost Li-ion battery with recyclable organic cathode and anode, self-standing graphite electrodes are also prepared. Graphite is the most common anode material used in Li-ion batteries^73^. With a theoretical capacity of 372 mAh·g^−1^, which corresponds to that of stage-I compound LiC_6_, graphite has remained a common choice for anodes in Li-ion batteries^74^. Section SI10 (Supporting Information) presents the electrochemical performances of different graphite electrodes made of pristine SO_3_H-modified graphite powders (Figs. SI11 and SI12). The self-standing anode is firstly combined with the LFP, and the full LFP/graphite battery is assembled with an excess capacity for the anode. The charge/discharge profile of the first cycle at C/24 of the corresponding battery is presented in Fig. 16a. A flat plateau at approximately 3.25 V is obtained with a small polarization, and the first discharge delivered is approximately 125 mAh·g^−1^. The overall potential is more interesting than that of the LFP/LTO system presented earlier; however, the specific capacity is lower because of the irreversible consumption of lithium by the graphite anode. As shown in Fig. 16a, this strong irreversibility results in a Coulombic efficiency of only 76% for the first cycle, but it practically reaches 100% in the succeeding cycles at 1 C. A long-term cycling experiment is also performed for 300 cycles at a cycling rate of 1 C. Figure 16b presents the stability of the LFP/graphite battery. After 300 charge/discharge cycles, the specific capacity is approximately 88 mAh·g^−1^, which corresponds to 85% of the initial discharge capacity obtained at 1 C. Although the Coulombic efficiency remains constant at approximately 100% during the entire experiment, a gradual capacity fading is evident probably because of the cathode and anode, as shown in Figs. 13 and SI12. In contrast, the all-paper LFP/graphite battery fabricated by Leijonmarck *et al*. exhibits poor cyclability^31^. In fact, after 300 charge/discharge cycles at 1 C, approximately 50 mAh·g^−1^ is obtained, which corresponds to a retention capacity of ~45%.

### Electrochemical performance of complete PTCDA/graphite batteries

**Figure 17:**
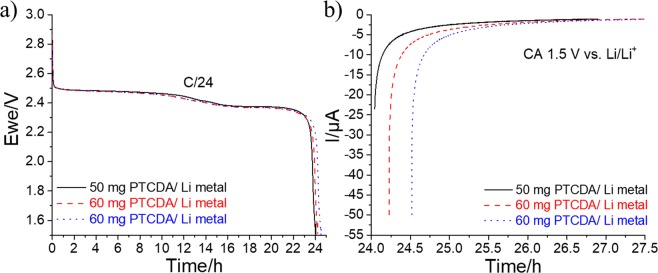
(**a**) Initial discharges obtained at a constant current of C/24 from OCP to 1.5 V versus Li/Li^+^ for three PTCDA/Li metal batteries. (**b**) Chronoamperometry experiment is directly performed at the end of discharge. The potential is fixed to 1.5 V versus Li/Li^+^ for 3 h.

An all-organic prototype PTCDA/graphite battery without transition metal is prepared and electrochemically tested. It is among the first self-standing batteries that is composed of organic redox molecules and biodegradable components reported in literature. The objective of this experiment is to demonstrate the concept of a battery that is both inexpensive and biodegradable. First, a prelithiation step is necessary, however, because the PTCDA molecule is in its oxidized form. To this end, the PTCDA self-standing film is assembled with a lithium counter electrode in a coin cell. Figure 17a shows the initial discharges of up to 1.5 V vs. Li/Li^+^ of three PTCDA/Li batteries made of 50 and 60 mg of organic cathode material. To achieve the complete lithiation of PTCDA, the potential is maintained at 1.5 V for 3 h (chronoamperometry experiment in Fig. 17b). The coin cell is disassembled, thereafter, and the prelithiated PTCDA cathode is recovered to be assembled with a graphite anode.

**Figure 18:**
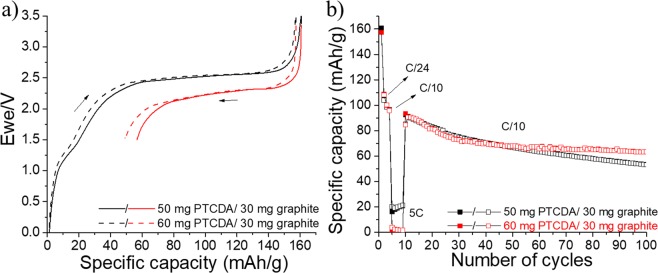
(**a**) Initial charges (— and ‒ ‒ ‒) and discharges ( and ) obtained at a constant current of C/24 between 1.5 and 3.5 V versus graphite of two prelithiated PTCDA/graphite batteries. A paper Kodoshi separator is used, and an excess anode capacity is employed. (**b**) Corresponding long cycling experiments are performed at a C/10 rate. The occurrence of a rapid formation is observed at 5 C. Full and empty characters represent charges and discharges, respectively.

The initial charges (— and ‒ ‒ ‒) and discharges ( and ) obtained at a constant current of C/24 between 1.5 and 3.5 V versus graphite of two prelithiated PTCDA/graphite batteries are presented in Fig. 18a. For the PTCDA, the first charge is higher than the theoretical capacity of 137 mAh·g^−1^. It reaches 160 mAh·g^−1^ because of the formation of SEI at the graphite anode surface. In fact, anodes in general^75^, especially graphite anodes^76^, sustain high first-cycle active lithium losses (ALL), which results from lithium-consuming parasitic reactions, such as the formation of an SEI^77^. In particular, carbons and graphites with high surface areas result in more SEI formations and thus higher ALL^78^. In general, the loss of active lithium resulting from its consumption in the positive electrode material permanently reduces the available energy. When the PTCDA/graphite battery is discharged, a small quantity of lithium is irreversibly consumed by the anode, and only 104 and 108 mAh·g^−1^ are obtained when the cathode is made of 50 and 60 mg of PTCDA, respectively. The long-term cycling experiments at C/10 for these batteries are shown in Fig. 18b. As observed in graphite electrodes (Fig. SI12), a continuous capacity loss upon cycling occurs in complete PTCDA/graphite batteries mainly because of the graphite anode’s consumption of lithium. A slight improvement is observed when the mass of prelithiated PTCDA increases because of the higher amount of available lithium. For this reason, future works will focus on the optimization of cathode/anode active mass ratios. Additional tests (e.g., prelithiation of anode) that could lead to the realization of a full organic battery with a stable capacity of approximately 120 mAh·g^−1^ should be planned.

## Conclusion

In the present work, an alternative method for fabricating Li-ion electrodes inspired of the paper-making process is proposed. The use of aluminum/copper current collectors, costly binders, and harmful solvents (NMP) are avoided in this fabrication method. These are replaced with non-modified cellulose, which is an abundant, natural, and low-cost polymer. The simple and rapid process is easily scalable and only uses water as solvent to prepare a suspension composed of redox active material, conductive carbon, and cellulose fibers. After successive filtrations, a self-standing electrode film is formed. It is made of an approximately 20-µm thick strong carbon current collector over which the electrode composite (cathode/anode material and carbon) is deposited. Water-soluble carbons and LFP are also produced to reduce the preparation time and facilitate the dispersion of electrode materials in water. The better repartition of these carbons in the electrode results in an improved electrochemical performance. Flexible LFP, PTCDA, graphite, and LTO electrodes with good mechanical resistance are obtained. The electrodes remain intact after punching, cycling, and opening of cells. The electrochemical performances of auto-supported electrodes are similar to those generally reported for LFP, PTCDA, graphite, and LTO casted on metal current collectors according to the conventional doctor blade casting method. The results of long cycling experiments conducted on full LFP/LTO and LFP/graphite batteries indicate that these cells have a remarkably good stability with high specific capacities. An all-organic prototype PTCDA/graphite battery without a transition metal is prepared and electrochemically tested. It is among the first self-standing batteries composed of organic redox molecules and biodegradable components reported in literature. Although the discharge capacity is relatively low, and the cyclability is not considerably stable, the concept of a battery that is both inexpensive and biodegradable is demonstrated. Further experiments, such as the optimization of the balance between cathode and anode masses and anode prelithiation, will be planned to achieve the fabrication of a full organic battery with a stable capacity of approximately 120 mAh·g^−1^. Moreover, organic cathode materials with a higher redox potential or higher specific capacity (e.g., 2,3-diamino-1,4-naphthoquinone (250 mAh·g^−1^))^79^ will be tested. In continuing this project, efforts will be focused on recycling electrodes that utilize a green process. A second-life battery made of recycled materials will be assembled and tested.

## Supplementary information

Supplementary information.
