# Supplementary material for: Toward Low-Cost All-Organic and Biodegradable Li-Ion Batteries
Source: Sci Rep. 2020 Mar 2;10:3812. doi: 10.1038/s41598-020-60633-y (PMC7052225; doi:10.1038/s41598-020-60633-y)
Supplement: Supplementary file 1 — Supplementary information. [file 41598_2020_60633_MOESM1_ESM.pdf]

SUPPORTING INFORMATION

Toward Low-Cost All-Organic and Biodegradable Li-Ion Batteries

N. Delaporte‡, G. Lajoie‡, S. Collin-Martin‡, K. Zaghib‡*

‡ Hydro-Québec, Center of Excellence in Transportation Electrification and Energy Storage, Varennes, Québec, J0L 1N0, Canada.
* zaghib.karim@hydro.qc.ca

SI1) Cost estimation of the new method of electrode fabrication

The method described in this manuscript is particularly interesting to reduce the cost of electrode fabrication. Firstly, it is known that cathode raw materials costs as well as processing costs represent almost 31% of the price of a full cell (i.e. LFP/graphite cell) [1]. The second non-negligible contribution is coming from separator (e.g. Celgard) and represents 24% of the total price. Another recent study evaluated than composite electrode materials, current collectors, separator and electrode processing represent about 62% of the cost of a Li-ion battery pack [2]. To reduce the costs, cathode fabrication need to be revisited and cheaper materials have to be selected. For these reasons, we chose a fabrication process that will only use water as solvent because it is the most abundant solvent, low-cost and green liquid. Process costs are lowered in this case but for a recyclability aspect, aluminum current collector has to be also removed since industrial methods for its recycling are neither economical nor energy efficient [3]. Inspired from paper making process, our fabrication method used natural cellulose as binder and support of the electrode. In comparison to conventional binder used in battery (i.e. PVDF) the price of this cellulose is more than 60 times lower (see Table SI1 for prices of cathode materials). In addition, this binder is eco-friendly, abundant and can be easily recycled at this end of battery life. The challenge of the new method, was to generate a compact and electrically conductive layer on a side of the electrode that will act as current collector. To reduce again the cost of fabrication, this layer should be fabricated in the same time than the cellulose film. Carbon fibers were selected as potential candidate due to their good mechanical properties and electronic conductivity. However, the bad dispersion of this carbon in water induced a step of surface functionalization to increase its affinity with the solvent and to create a carbon mat during the rapid filtration process. The cost for the surface modification of the VGCF carbon is not expected to be high since the precursors for the synthesis are relatively cheap and widely available. Our cost estimation for the functionalized VGCF-COOH carbon is about 23 USD (see Table SI1). Due to the absence of heavy and costly Al current collector, higher amount of carbon can be permitted for the composition of the cathode that consequently increases the electronic conductivity of the cathode film. In fact, for a similar electrode loading of about 11 $mg.cm^{-2}$ (see Table SI2), the quantity of carbon for the cathode (including Al current collector) made following the standard method is about 2.7 wt.% while the self-standing film was composed of 17 wt.% (the half for the carbon current collector). Even if the price of modified-VGCF carbon is quite high, the estimated price of solid materials per square meter of electrode

was calculated to 1.8 USD for the new method of fabrication in comparison to 3.1 USD for the conventional. Table SI2 shows that if the same amount of LFP is used in both the conventional and the self-standing electrodes (i.e. 10 mg) then the cost is still more attractive for the new method of fabrication (2.5 USD).

Most importantly, solvents and processing costs have not been taking into account for this calculation and are assuredly more advantageous for our fabrication method (cheap solvent, easy drying, paper making process well established and very rapid). In addition, as said earlier, the cost of conventional separator represents almost 24% of the battery cost [2] and the possibility to fabricate a paper separator on the top of the electrode further reduces the overall price of our method. Finally, organic electrodes (e.g. PTCDA) should lower the cost of battery manufacturing because organic materials can be prepared from natural products and biomass in addition to make electrodes totally recyclable.

Table SI1. Prices of cathode materials, precursors for surface modification of carbon, solvents and current collector employed following the conventional or the new fabrication method.

Cathode component	Material price (USD)	References
**Cellulose pulp**	< 0.45 $/kg	[4]
**Tert-butyl nitrite**	1-5 $/kg	[5]
**Aminobenzoic acid**	1-2 $/kg	[6]
**Carbon**	7.15 $/kg	[7]
**VGCF carbon**	20 $/kg	[8]
**Modified VGCF**	23 $/kg	Our cost estimation*
**PVDF binder**	27.6 $/kg	[7]
**NMP solvent**	3.2 $/kg	[7]
**Water solvent**	<0.1 $/kg	[9]
**Cathode material**	21 $/kg	[7]
**Al current collector**	0.8 $/m^2	[7]

* *Based on precursors price (higher limit) and labor (15% increase).*

Table SI2. Estimated price of solid materials (per m^2 of electrode) for an LFP cathode made following the conventional and the new fabrication method and taking into account the percentage of the different components. For comparison, two loadings are presented for our method.

	**Conventional method (11 mg/cm^2)**		**Our method (11 mg/cm^2)**		**Our method (14.65 mg/cm^2)**	
	% material*	Mass of material	% material	Mass of material	% material	Mass of material
**LFP**	62	10 mg	58	6.38 mg	68	10 mg
**Carbon**	2.7	0.44 mg	17	1.87 mg	13	1.86 mg
**Binder**	3.4	0.55 mg	25	2.75 mg	19	2.79 mg
**Al current collector**	31.9	5.12 mg	0	0	0	0
**Estimated price of solid materials (per m^2 of electrode)**	**3.1 USD**		**1.8 USD**		**2.5 USD**	

Electrode composition based on reference [1]: 91% LFP, 4% carbon and 5% binder.

SI2) SEM analyses of LTO self-standing film

Figure SI1 shows the SEM images of a) FB-rich side and c) carbon-rich side of an LTO self-standing film made of VGCF–COOH carbon. The corresponding elemental mappings of Ti (blue) and C (red) are also provided. Figures 6 and 7 in the manuscript show different views (i.e., cross-section) of the same electrode film. The LTO material efficiently fills the pores of the cellulose mat, whereas the other side of the film remains intact and is only composed of carbon, i.e., the integrated carbon current collector.

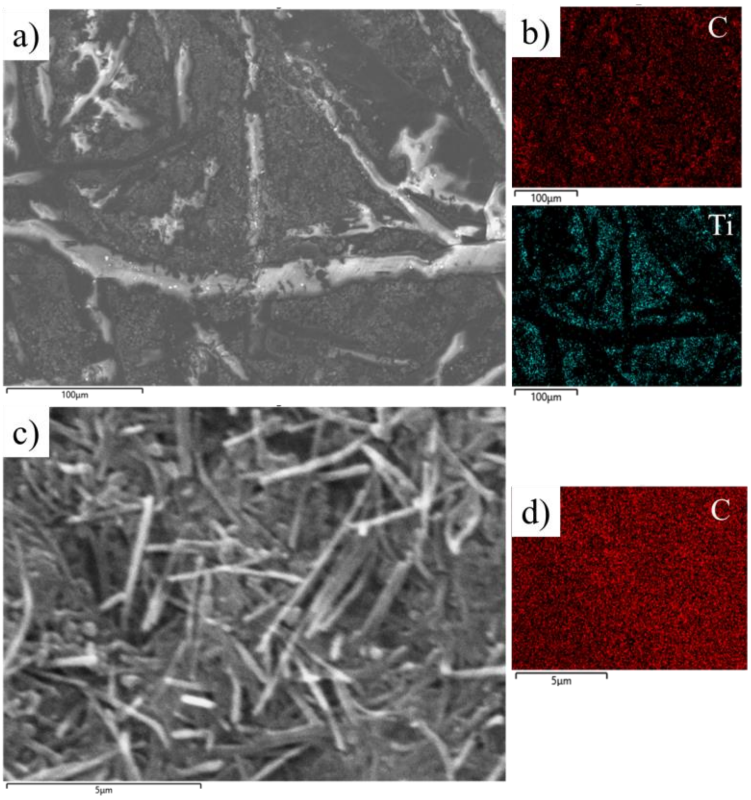

Figure SI1. SEM images of a) FB-rich side and c) carbon-rich side of an LTO self-standing film made of VGCF–COOH carbon. b, d) Corresponding elemental mappings of Ti (blue) and C (red).

SI3) Effect of electrode calendering on performances

The effect of calendering at different temperatures is investigated. The cyclic voltammogram and rate capability of the same electrode pressed at 25 and 50 °C are presented in Figure SI2. First, as clearly demonstrated by the cyclic voltammetry experiments (Figure SI2a), excellent reproducibility is noted in the two batteries made of the same electrodes. Regardless of whether the electrode is composed of CNT, Denka, or VGCF, the reproducibility is perfect. Second, a small capacity can be recovered for all electrodes after calendering at 50 and 80 °C, as shown in Figure SI2 and Figure 9 in the manuscript, respectively. This may be the result of a better electrical contact between the active material and carbon inside the film.

Finally, and most interestingly, the thickness of the self-standing film decreases with the increase in the calendering temperature. At 25 °C, an approximately 100-µm thick film is obtained, whereas thicknesses of ~80 and ~70 µm are measured at 50 and 80 °C, respectively. Based on the results of the different electrochemical tests performed, the type of carbon utilized has no significant effect on film thickness. After calendaring, the thickness appears to be governed by the amount of cellulose fibers used.

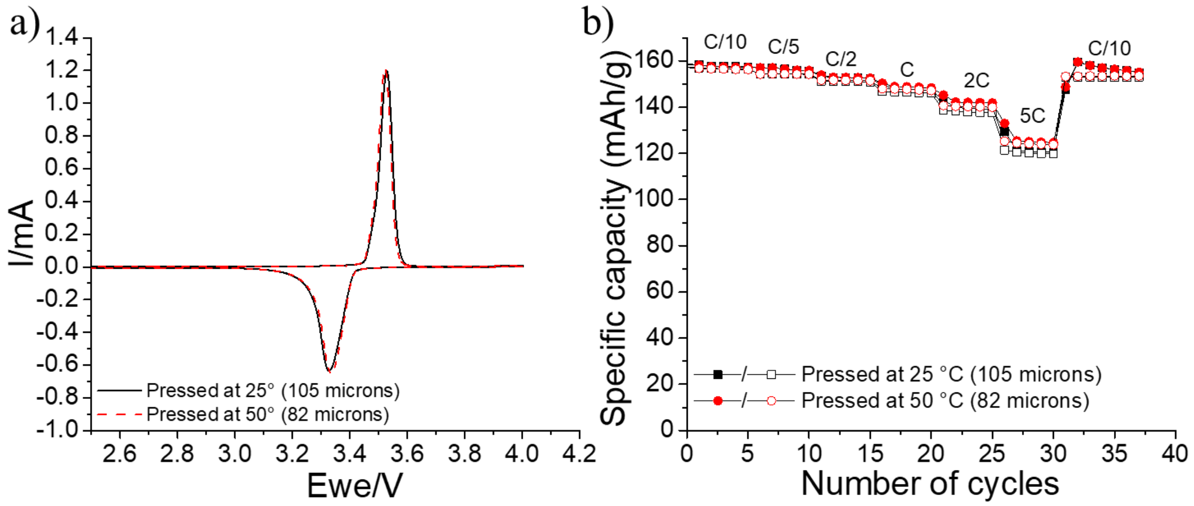

Figure SI2. a) Cyclic voltammetry experiments performed at a scan rate of 0.03 mV·s^{-1} for two LFP electrodes containing a mixture of VGCF–COOH and CNT–COOH carbons and calendered at 25 (—) and 50 °C (– – –), respectively. b) Comparison of rate capabilities for the same electrode. Full and empty characters represent charges and discharges, respectively.

SI4) Impact of carbon used in the LFP electrode formulation on electrochemical performance

The influence of the carbon type on the electrochemical performance is also evaluated. Denka and CNT carbons are also treated for easy dispersion in water because better results are obtained with the modified VGCF. In the electrode composition, 25% of the quantity of VGCF is replaced by another type of carbon (e.g., Denka or CNT). Figure SI3 compares the rate capabilities of LFP electrodes made of different mixtures of modified carbons. Following the new method of fabrication, it is evident that the use of Denka carbon is not a good choice to prepare Li-ion films. A clear improvement is observed when Denka is replaced with CNT. In fact, when Denka is used, the specific capacity obtained at 1C is approximately 140 mAh·g^{-1}, whereas for electrodes that contain the modified CNT, the same capacity is achieved at 2C. Regarding the use of CNT, a slight improvement is observed compared to an electrode that is entirely made of VGCF. The high cost of such a carbon, however, has to be considered and is probably not suitable for the new low-cost fabrication method.

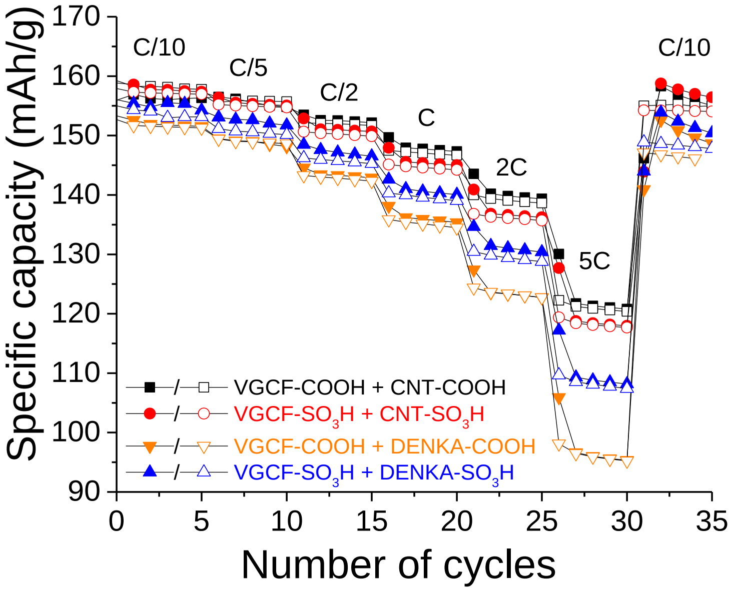

Figure SI3. Comparison of rate capabilities of LFP electrodes containing the following mixtures: VGCF–COOH and Denka–COOH (▼); VGCF–SO_3H and Denka–SO_3H (▲); VGCF–COOH and CNT-COOH (■); VGCF–SO_3H and CNT–SO_3H (●). Full and empty characters represent charges and discharges, respectively.

SI5) Impact of carbon used in the LTO electrode formulation on electrochemical performance

As for LFP cathodes, 25% of modified-VGCF was replaced with grafted Denka and CNTs. The electrochemical performances of such electrodes are represented in Figure SI4. The cyclic voltammetry experiments revealed a redox peak more intense and sharp when CNT-COOH was used (– – –) rather than Denka-COOH (····) or only VGCF-COOH (—). This was also confirmed by cycling experiments at various C-rates (Figure SI4b). Approximately, 120, 105, and 95 $mAh.g^{-1}$ were obtained at 5C for LTO electrodes made of CNT-COOH and VGCF-COOH, only VGCF-COOH, and Denka-COOH and VGCF-COOH, respectively. Exactly the same results were obtained when -aryl-COOH groups were changed for -aryl-SO_3H (not shown). As concluded with Figure SI3 for cycling of LFP electrodes, the use of Denka carbon was not a good choice to make LTO electrodes according to the new process of fabrication. On the contrary, adding a small amount of modified CNTs clearly improved the electrochemical performance, in particular at high C-rates.

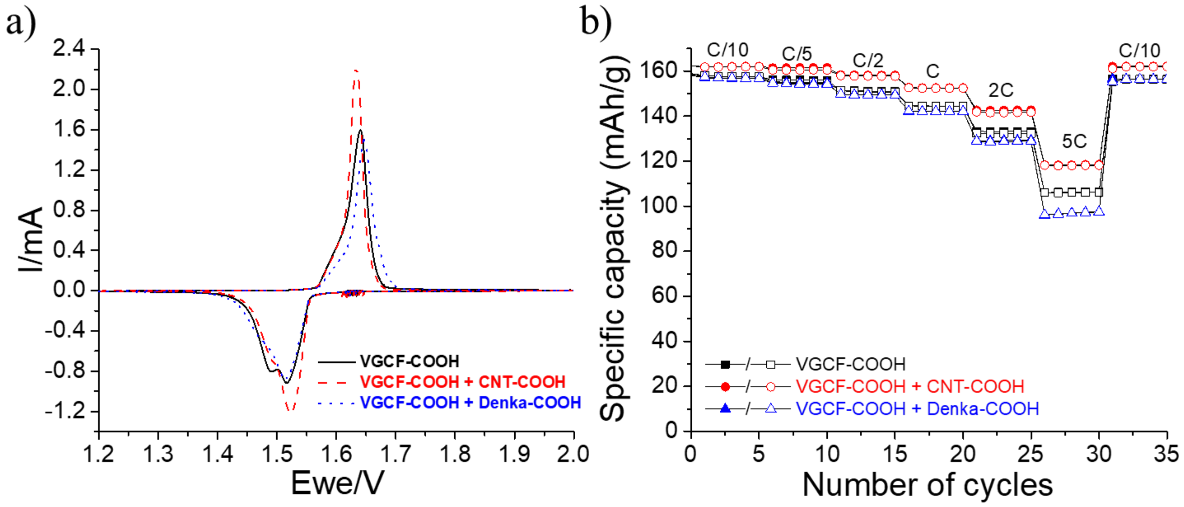

Figure SI4. a) Cyclic voltammetry experiments realized at a scan rate of 0.03 mV.s^{-1} for three LTO electrodes containing only VGCF-COOH (—), a mixture of VGCF-COOH and CNT-COOH (– – –), and a mixture of VGCF-COOH and Denka-COOH (····). b) Comparison of rate capabilities of the three electrodes. Solid and open symbols are used to represent charge and discharge cycles, respectively.

SI6) Long cycling experiment for LTO/Li cell

The LTO/Li coin cell was disassembled after 300 charge/discharge cycles at a C/2 rate. The optical photographs presented in Figure SI5 shows a) the Celgard separator, b) the self-standing LTO electrode, and c) the lithium electrode after charge-discharge cycles. It is evident that the self-standing electrode remained intact after cycling without any dissolution of the active material or carbon in the electrolyte.

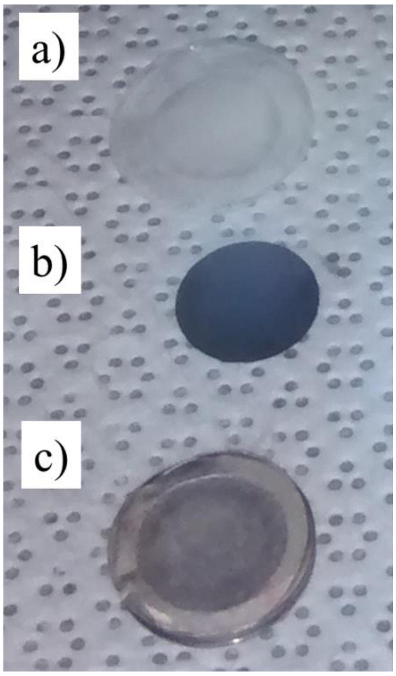

Figure SI5. Optical photography showing a) a Celgard separator, b) a self-standing LTO electrode, and c) a lithium electrode after disassembling a cell that has undergone 300 charge/discharge cycles at a constant current of C/2 between 1.2 and 2.5 V versus Li/Li^{+}.

SI7) Impact of separator on electrochemical performance of full LFP/LTO batteries

Figure SI6 shows the rate capabilities of two LFP/LTO batteries with same amounts of the active material for both electrodes but with different separators. Interestingly, the discharge capacities obtained were higher when a Kodoshi paper separator (●) was used instead of a conventional Celgard (■). An additional 10 $mAh.g^{-1}$ was obtained for the all-paper battery at each C-rate ranging from C/24 to 2C in comparison to the cell assembled with a Celgard separator. Thus, discharge capacities of 100 and 115 $mAh.g^{-1}$ were delivered at 5C for the battery using the Celgard and the paper separator, respectively. These performances are encouraging for the development of all-paper Li-ion batteries and lowering the cost of the energy storage devices by replacing Celgard with a paper separator.

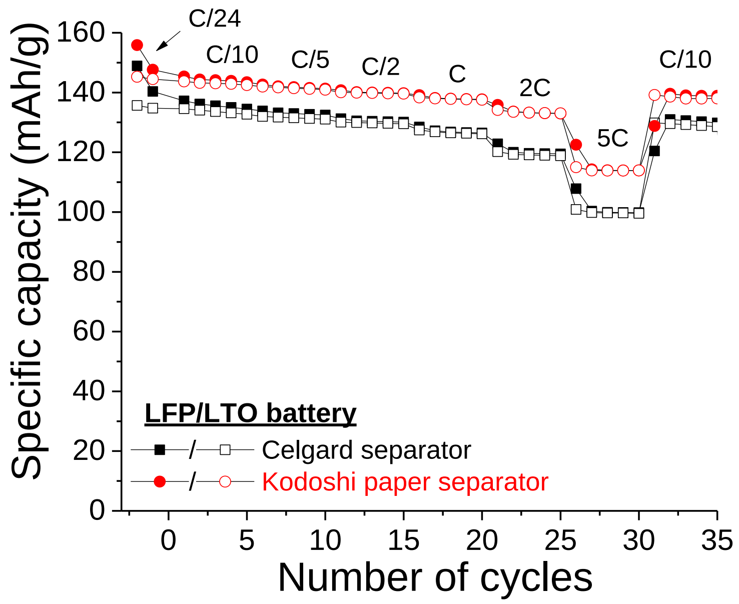

Figure SI6. Comparison of rate capabilities of the LFP/LTO battery (LFP/LTO mass ratio = 1) with those of Celgard (■) and paper Kodoshi (●) separators. Solid and open symbols are used to represent charge and discharge cycles, respectively.

SI8) Long-term cyclability of full LFP/LTO batteries

Figure SI7 shows the cyclability of the LFP/LTO full cell with 40 mg of LFP and LTO. High coulombic efficiency of ~99.9% was obtained over 1000 charge/discharge cycles performed at a 2C rate, similar to that for the cell with a higher active mass loading of 70 mg (Figure 13 in the manuscript). After 1000 cycles, the capacity retention was approximately 82.9% for the electrodes with 40 mg of active materials. It increased to 91.6% for the cell with 70 mg of LFP/LTO (Figure 13).

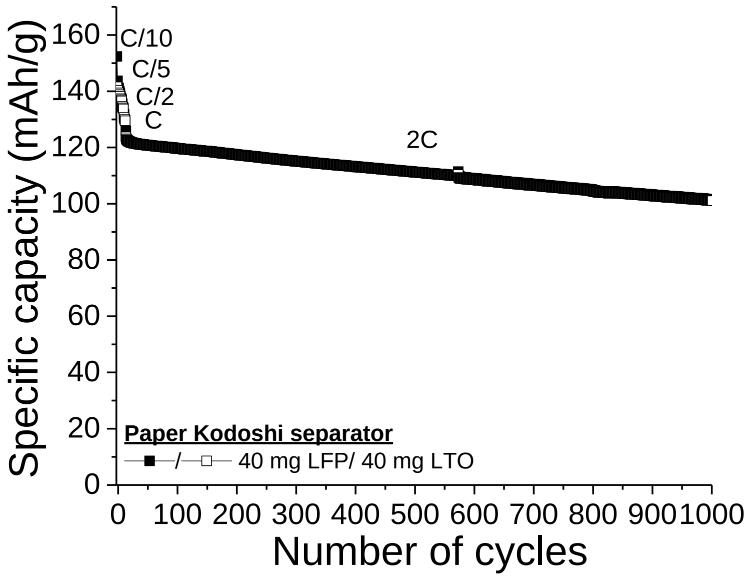

Figure SI7. Long cycling experiment realized at a constant charge/discharge current of 2C between 1.0 and 2.5 V versus LTO for the LFP/LTO battery with 40 mg of LFP and LTO. Paper Kodoshi separator was used. Solid and open symbols are used to represent charge and discharge cycles, respectively.

Figure SI8a presents capacity retention over 200 charge/discharge cycles for same battery compositions but assembled with the Celgard (□) and Kodoshi paper separators (○). As seen in Figure SI6, the use of a paper separator led to higher specific capacities. Approximately 140 and 130 $mAh.g^{-1}$ capacities were obtained for the first cycle at C/2 when the paper and Celgard separators were used, respectively. After 200 cycles, approximately 92% of the initial discharge capacity was recovered. The paper separator did not enhance the capacity retention, although the specific capacities were better. A gradual loss of capacity was observed; however, it was less pronounced when higher active mass loadings were used as shown in the long cycling experiments in Figure SI8b (also compare Figure 13 in the manuscript with Figure SI7). In fact, when 40, 60, and 70 mg of LFP were used, capacity retentions of 89.6, 90.8, and 91.4% were obtained, respectively.

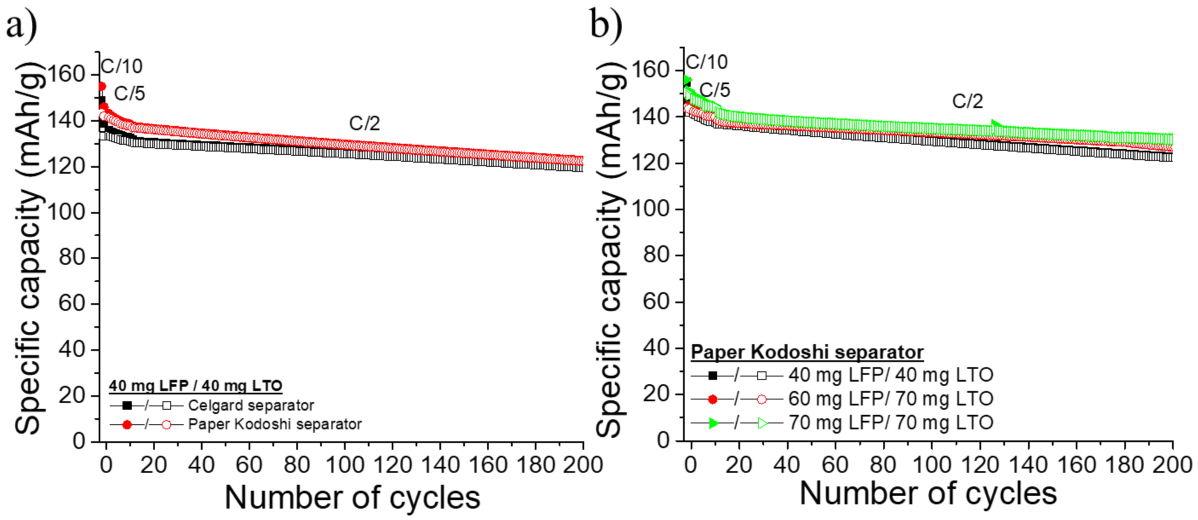

Figure SI8. Long cycling experiments realized at a constant charge/discharge current of C/2 between 1.0 and 2.5 V versus LTO. a) Stability for the same LFP/LTO battery is compared when Celgard (□) and paper Kodoshi (○) separators are used. b) Different amounts of LFP and LTO are utilized with LFP/LTO mass ratios = 1 and ~0.85. Paper Kodoshi separator was used for all electrodes. Solid and open symbols are used to represent charge and discharge cycles, respectively.

SI9) Electrochemical performance of PTCDA electrodes

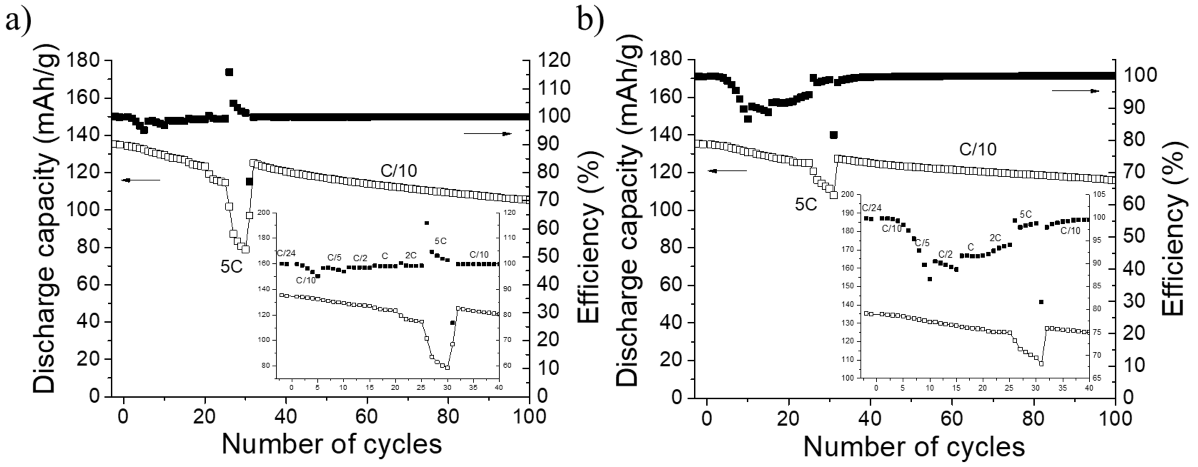

Figure SI19. Long cycling experiments performed at a constant charge/discharge current of C/10 between 1.5 and 3.5 V versus Li/Li^+. Two PTCDA electrodes (40 mg), one composed of a) VGCF–COOH, and the other of b) a mixture of VGCF–SO_3H and CNT–SO_3H, are compared. Full and empty characters represent the Coulombic efficiency (%) and discharge capacity, respectively. The inset shows the rate capability at different C rates before the long cycling experiment.

The cyclability of PTCDA electrodes are evaluated by long-term cycling experiments at C/10 after several cycles at different C rates ranging from C/24 to 5C. Figure SI9 presents the capacity retention over 100 charge/discharge cycles for the same composition of electrode except that the results presented in Figure SI9b are obtained when a small quantity of CNT–

SO_3H is utilized. PTCDA molecule has a low electrical conductivity, and the use of a high quantity of conductive carbon in the composition of the electrode is necessary to achieve good electrochemical performance. When highly conductive CNT–SO_3H is utilized (Figure SI9b), approximately 110 mAh·g^{-1} is achieved at 5C, whereas 80 mAh·g^{-1} is delivered when the electrode is only composed of VGCF–COOH as carbon source (Figure SI9a). Another aspect that is observed is the Coulombic efficiency (%), which distinctly decreases with the number of cycles. A zoom image of the first 40 cycles of each cycling is shown in the insets of Figure SI9. In both cases, after 4 cycles at a low C rate, the Coulombic efficiency gradually decreases to achieve an instance of 85% after 12 cycles for the battery containing a small amount of CNT–SO_3H (Figure SI9b). This observation is already reported in literature and is associated with the high solubility of PTCDA molecule in the electrolyte. In a recent study, the authors assumed that the phenomenon is caused by the solubilization of anhydride monomer in the electrolyte that crosses the separator and subsequently deposits on the lithium anode surface, leading to a loss of the active material [10]. Sharma *et al.* reported a low-capacity retention corresponding to 37% of the initial discharge capacity (132 mAh·g^{-1}) of a PTCDA electrode (on an aluminum foil) after only 50 cycles at 50 mA·g^{-1} [11]. A self-standing film made of PTCDA/3,4,9,10-perylenetetracarboxylic diimide (PTCDI) mixture with CNTs is capable of delivering ~60 mA·g^{-1} when cycled under constant currents ranging from 50 to 2000 mA·g^{-1} and maintained at a low Coulombic efficiency of 95% during long cycling experiments for 500 cycles (with a constant curent of 50 mA·g^{-1}) [12]. The Coulombic efficiency is significantly improved with the increase in C rate, as shown in the inset of Figure SI9b. After five cycles at 5C, the long-term cycling at C/10 has started, and interestingly, the Coulombic efficiency has reached ~100%. A relatively good stability over 100 cycles is observed as 84 and 91% of the initial discharge capacity at C/10 at the end of the experiment for electrodes made of VGCF–COOH (Figure SI9a) and the mixture of VGCF–SO_3H and CNT–SO_3H (Figure SI9b), respectively. The incorporation of CNT–SO_3H results in a better stability, which is frequently caused by the higher electrical conductivity of the cathode film.

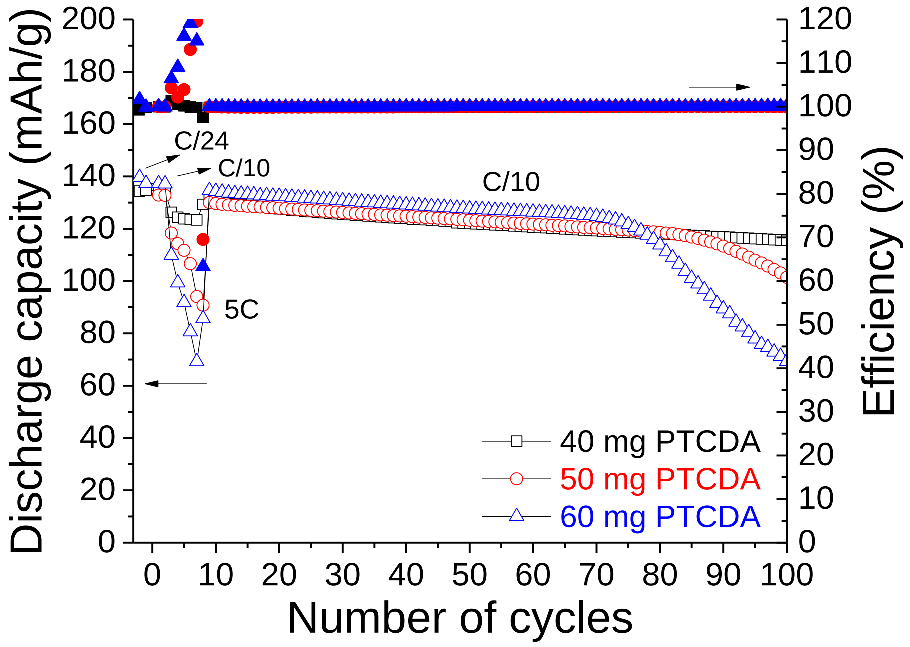

Figure SI10. Long cycling experiments realized at a constant charge/discharge current of C/10 between 1.5 and 3.5 V versus Li/Li^+. Three PTCDA electrodes with different active material loadings and containing a mixture of VGCF-SO_3H and CNT-SO_3H are compared. Solid and open symbols represent the coulombic efficiency (%) and discharge capacity, respectively. Before the long cycling experiment, a formation at 5C was rapidly effected.

The cyclabilities of PTCDA electrodes, made with 40, 50, and 60 mg of active material, were investigated. Figure SI10 shows a comparison of the cyclabilities at C/10 over 100 cycles for three PTCDA electrodes with different active mass loadings. As shown in Figure SI9, when the loading of active material was increased, the discharge capacity obtained at high C-rates, and particularly at 5C, sharply decreased because of the higher resistance of the cathode film [13]. Approximately, 91, 92, and 92% of the initial discharge capacities at C/10 were obtained after 70 charge/discharge cycles for the electrodes made with 40, 50, and 60 mg of PTCDA, respectively. However, after these 70 cycles, the specific capacity suddenly dropped for self-standing films with 50 (Figure SI10, ○) and 60 mg (Figure SI10, Δ) of active material. As suggested by the stability experiment in Figure 15, the gradual loss of capacity is mainly related to the degradation of the Li metal anode [14].

SI10) Electrochemical performance of graphite electrodes

Long cycling experiments at the rate of C/10 were realized for self-standing graphite electrode films made with 40 mg of anode material and with VGCF-SO_3H (□) and a mixture of VGCF-SO_3H and CNT-SO_3H (○) as conductive additives. The corresponding long-term cycling tests are shown in Figure SI11. As generally observed for graphite, during the first cycle, the coulombic efficiency is higher than 100% and reached 120% and 145% when VGCF-SO_3H (□) and a mixture of VGCF-SO_3H and CNT-SO_3H (○) was used, respectively. The use of modified CNTs significantly increased the specific surface area of the anode composite

electrode and led to more significant electrolyte degradation and consumption of lithium. However, the higher electrical conductivity of the self-standing film made with CNTs induced a better cycling stability and delivered an additional 75 mAh.g^{-1} in comparison with the electrode without CNTs. Although the electrochemical performance is interesting, the graphite anode made with CNTs (○) cannot be used for a full Li-ion battery owing to its high irreversibility. A gradual loss of capacity upon cycling was observed for both electrodes, but more important for the anode composite without CNTs. This behavior is mainly related to the overall electrical conductivity of the electrode as shown Figure SI12.

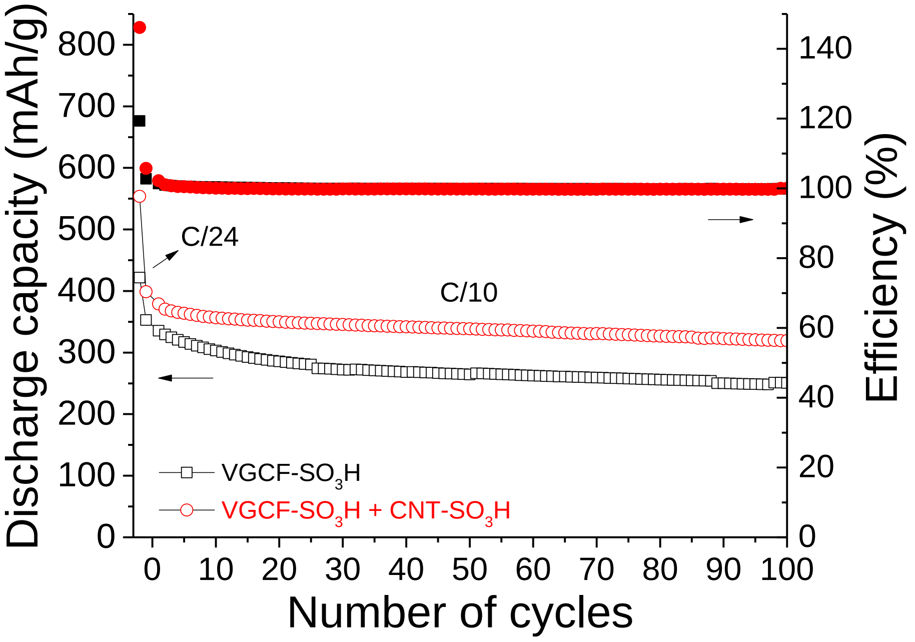

Figure SI11. Long cycling experiments realized at a constant charge/discharge current of C/10 between 0 and 1.5 V versus Li/Li^{+}. Two graphite electrodes, one composed of VGCF-SO_3H (□) and the other of a mixture of VGCF-SO_3H and CNT-SO_3H (○), are compared. Solid and open symbols represent the coulombic efficiency (%) and discharge capacity, respectively.

In Figure SI12a, two graphite electrodes with different loadings (30 and 40 mg of active material) are compared. As suggested by the results in Figure SI11, capacity fading is related to the resistance of the self-standing film. Higher the amount of graphite used for the fabrication, higher the resistance of the film. Thus, with 30 mg of graphite, the initial discharge capacity at C/10 was about 380 mAh.g^{-1}, close to the theoretical capacity of graphite. The loss of capacity upon cycling is less pronounced than for the electrode made with 40 mg of graphite (○). Figure SI12b presents the cyclabilities of three electrodes with modified graphite-SO_3H. Interestingly, using the functionalized carbon led to improved performances. First, the discharge capacity for a similar active material loading as for unmodified graphite electrodes (see □ and ○) was significantly higher, particularly when 40 mg of active material was used. The first discharge at C/10 gave 335 and 405 mAh.g^{-1} for electrodes with graphite and graphite-SO_3H, respectively. Furthermore, increasing the graphite-SO_3H mass up to 50 mg (Δ) led to

improved cyclability and higher specific capacities than those obtained with 40 mg of graphite. Second, the -SO_3H groups attached to the carbon surface increase the overall capacity of the battery, because a specific capacity of more than 400 mAh.g^{-1} was obtained over several cycles. These groups may contribute to the storage of lithium and the increase in its ionic conductivity. A similar behavior was recently reported after the modification of the $LiFePO_4$ surface with Li^+-conductor groups [15]. The cyclability and coulombic efficiency were definitively better than those obtained with graphite/microfibrillated cellulose nanoparticles (MFC) [16] and graphite/Kraft bleached hardwood cellulose fiber [17] composite electrodes. Finally, the coulombic efficiency was slightly lower during the first cycle at C/24 when the modified carbon was used.

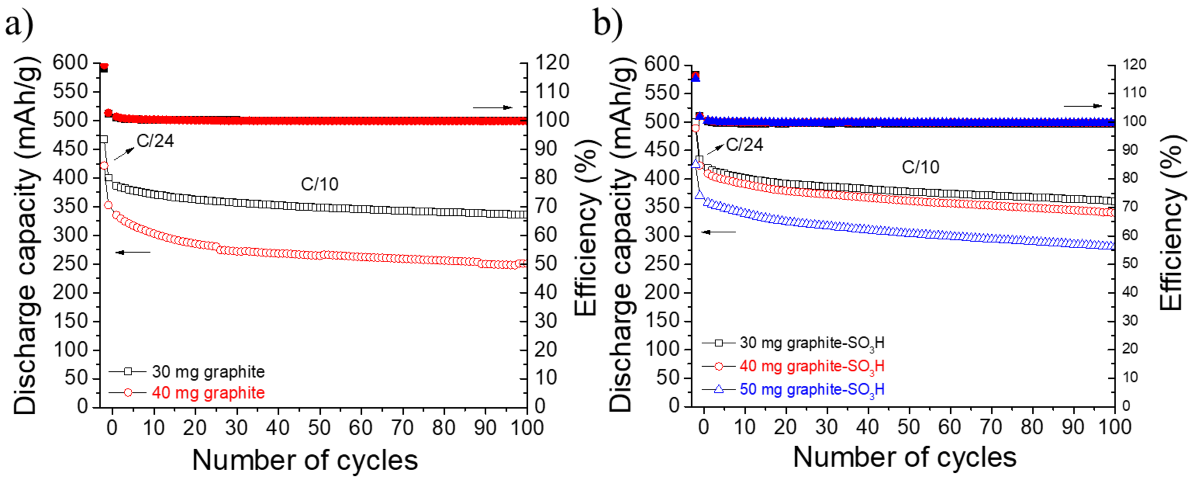

Figure SI12. Long cycling experiments realized at a constant charge/discharge current of C/10 between 0 and 1.5 V versus Li/Li^+. Several electrodes composed of VGCF-SO_3H as the carbon additive are compared. The impacts of the use of a) graphite and b) graphite-SO_3H are shown. Solid and open symbols represent the coulombic efficiency (%) and discharge capacity, respectively.

[1] Wentker, M., Greenwood, M. & Leker, J. A Bottom-Up Approach to Lithium-Ion Battery Cost Modeling with a Focus on Cathode Active Materials. *Energies* **12**, 504 (2019).
[2] Wood III, D. L., Li, J. & Daniel, C. Prospects for reducing the processing cost of lithium ion batteries. *J. Power Sources* **275**, 234-242 (2015).
[3] Gaines, L. The future of automotive lithium-ion battery recycling: Charting a sustainable course. *SM&T* **1-2**, 2-7 (2014).
[4] Brian McClay & Associés inc. Current lumber, pulp and panel prices. https://www.nrcan.gc.ca/our-natural-resources/domestic-international-markets/current-lumber-pulp-panel-prices/13309.
[5] https://www.aecochemical.com/.
[6] https://ahlandy.en.alibaba.com/.
[7] Patry, G., Romagny, A., Martinet, S. & Froelich, D. Cost modeling of lithium-ion battery cells for automotive applications. *Energy Sci. Eng.* **3**, 71-82 (2015).
[8] Tibbetts, G. G., Lake, M. L., Strong, K. L. & Rice, B. P. A review of the fabrication and properties of vapor-grown carbon nanofiber/polymer composites. *Compos. Sci. Technol.* **67**, 1709-1718 (2007).

[9] Average monthly residential cost of water in the U.S. from 2010 to 2018 (in U.S. dollars). https://www.statista.com/statistics/720418/average-monthly-cost-of-water-in-the-us/.
[10] Wang, J., Wang, X. M., Li, H. F., Yang, X. W. & Zhang, Y. G. Intrinsic factors attenuate the performance of anhydride organic cathode materials of lithium battery. *J. Electroanal. Chem.* **773**, 22-26 (2016).
[11] Sharma, P., Damien, D., Nagarajan, K., Shaijumon, M. M. & Hariharan, M. Perylene-polyimide-Based Organic Electrode Materials for Rechargeable Lithium Batteries. *J. Phys. Chem. Lett.* **4**, 3192-3197 (2013).
[12] Yuan, C. *et al*. Free-standing and flexible organic cathode based on aromatic carbonyl compound/carbon nanotube composite for lithium and sodium organic batteries. *J. Colloid Interface Sci.* **517**, 72-79 (2018).
[13] Tenne, D. A. *et al*. Single crystals of the organic semiconductor perylene tetracarboxylic dianhydride studied by Raman spectroscopy. *Phys. Rev. B* **61**, 14564-14569 (2000).
[14] Delaporte, N., Wang, Y. & Zaghib, K. Pre-treatments of Lithium Foil Surface for Improving the Cycling Life of Li Metal Batteries. *Front. Mater.* **6**, Article number: 267 (2019).
[15] Delaporte, N. *et al*. Increasing the Affinity Between Carbon-Coated $LiFePO_4$/C Electrodes and Conventional Organic Electrolyte by Spontaneous Grafting of a Benzene-Trifluoromethylsulfonimide Moiety. *ACS Appl. Mater. Interfaces* **7**, 18519-18529 (2015).
[16] Jabbour, L. *et al*. Microfibrillated cellulose–graphite nanocomposites for highly flexible paper-like Li-ion battery electrodes. *J. Mater. Chem.* **20**, 7344-7347 (2010).
[17] Jabbour, L. *et al*. Aqueous processing of cellulose based paper-anodes for flexible Li-ion batteries. *J. Mater. Chem.* **22**, 3227-3233 (2012).
